# Doxorubicin-loaded liposomes surface engineered with the matrix metalloproteinase-2 cleavable polyethylene glycol conjugate for cancer therapy

**DOI:** 10.1186/s12645-023-00169-8

**Published:** 2023-03-07

**Authors:** Anis Askarizadeh, Mohammad Mashreghi, Elaheh Mirhadi, Farshad Mirzavi, Vahid Heravi Shargh, Ali Badiee, Seyedeh Hoda Alavizadeh, Leila Arabi, Mahmoud Reza Jaafari

**Affiliations:** 1 Nanotechnology Research Center, Pharmaceutical Technology Institute, Mashhad University of Medical Sciences, Mashhad, Iran; 2 Department of Pharmaceutical Nanotechnology, School of Pharmacy, Mashhad University of Medical Sciences, Mashhad, Iran; 3 Cardiovascular Diseases Research Center, Birjand University of Medical Sciences, Birjand, Iran; 4 Division of Pharmacy and Optometry, School of Health Sciences, Faculty of Biology, Medicine and Health, The University of Manchester, Manchester, UK

**Keywords:** Doxorubicin, Cationic liposome, Matrix metalloproteinase-2, Vascular targeting, Cancer

## Abstract

**Background:**

Colorectal cancer is one of the prominent leading causes of fatality worldwide. Despite recent advancements within the field of cancer therapy, the cure rates and long-term survivals of patients suffering from colorectal cancer have changed little. The application of conventional chemotherapeutic agents like doxorubicin is limited by some drawbacks such as cardiotoxicity and hematotoxicity. Therefore, nanotechnology has been exploited as a promising solution to address these problems. In this study, we synthesized and compared the anticancer efficacy of doxorubicin-loaded liposomes that were surface engineered with the 1,2-dioleoyl-sn-glycero-3-phosphoethanolamine-matrix metalloproteinase-2 (MMP-2) cleavable peptide-polyethylene glycol (PEG) conjugate. The peptide linker was used to cleave in response to the upregulated MMP-2 in the tumor microenvironment, thus exposing a positive charge via PEG-deshielding and enhancing liposomal uptake by tumor cells/vasculature. Liposomal formulations were characterized in terms of size, surface charge and morphology, drug loading, release properties, cell binding and uptake, and cytotoxicity.

**Results:**

The formulations had particle sizes of ~ 100–170 nm, narrow distribution (PDI ˂ 0.2), and various surface charges (− 10.2 mV to + 17.6 mV). MMP-2 overexpression was shown in several cancer cell lines (C26, 4T1, and B16F10) as compared to the normal NIH-3T3 fibroblast cells by gelatin zymography and qRT-PCR. In vitro results demonstrated enhanced antitumor efficacy of the PEG-cleavable cationic liposomes (CLs) as compared to the commercial Caelyx^®^ (up to fivefold) and the chick chorioallantoic membrane assay showed their great antiangiogenesis potential to target and suppress tumor neovascularization. The pharmacokinetics and efficacy studies also indicated higher tumor accumulation and extended survival rates in C26 tumor-bearing mice treated with the MMP-2 cleavable CLs as compared to the non-cleavable CLs with no remarkable sign of toxicity in healthy tissues.

**Conclusion:**

Altogether, the MMP-2-cleavable CLs have great potency to improve tumor-targeted drug delivery and cellular/tumor-vasculature uptake which merits further investigation.

**Supplementary Information:**

The online version contains supplementary material available at 10.1186/s12645-023-00169-8.

## Background

Colorectal cancer (CRC) is one of the most frequently occurred malignancies worldwide, which was recently estimated to rank as the second leading cause of cancer mortality (9.4% of the total cancer deaths) (Sung et al. 2021). Although new therapeutic strategies, including radiotherapy, surgery, and neoadjuvant/palliative chemotherapies have emerged during the last decades, the cure rates and long-term survivals of patients suffering CRC have changed little (Kuipers et al. 2015). The anthracycline, doxorubicin (Dox), is a broad-spectrum chemotherapeutic agent which is clinically used to treat a wide range of carcinomas, sarcomas and hematological cancers (van der Zanden et al. 2021). CRC cells are intrinsically resistant to Dox and require higher therapeutic doses, which can exceed its maximum tolerated dose, limiting its clinical application as adjuvant chemotherapy at advanced stages of the disease (Sonowal et al. 2017). Dox-induced cardiotoxicity and hematotoxicity are dose-dependent, highlighting the need to increase its tumor-targeted delivery and blood circulation half-life to decrease the overall administered dosage (Yao et al. 2019).

Nanotechnology is a promising tool in cancer therapy, formulating delivery systems that exploit the enhanced permeability and retention (EPR) effect for localized tumor accumulation and providing sustained release and enhanced in vivo half-life of desirable therapeutic payloads (Şen et al. 2021). Doxil^®^/Caelyx^®^ (PEGylated liposomal Dox) is the first FDA-approved biocompatible nanoparticle introduced in 1995 for passive tumor targeting (Barenholz 2012). Blood clearance and volume of distribution of Dox were drastically reduced when incorporated in PEGylated liposomes, significantly improving its therapeutic index while restricting toxicities in the clinic (Gabizon et al. 2003). Although PEGylation has generally been thought to shield nanoparticles from the mononuclear phagocyte system (MPS) after systemic administration and prolong their circulation lifetime, its surface steric hindrance may limit their interaction and uptake into the tumor cells (known as the PEG dilemma) (Pozzi et al. 2014). Correspondingly, a previous report investigating the effect of PEGylation on the accumulation of liposomal Dox in murine Lewis lung carcinoma could not detect a significant advantage in tumor drug delivery or enhanced therapeutic activity over non-PEGylated formulation (Parr et al. 1997). Hence, novel strategies such as tumor microenvironment-responsive and cleavable PEG coatings can potentially be applied to boost cellular uptake of nanoparticles at the malignant site (Fang et al. 2017).

Cationic liposomes (CLs) have been used as delivery platforms for selective drug delivery to solid tumors via their potential electrostatic interactions with overexpressed molecules, such as anionic phospholipids, and glycoproteins, proteoglycans of angiogenic endothelial cells in tumor vasculature and cellular membranes (Abu Lila et al. 2010; Dicheva et al. 2014). EndoTAG-1 as a proof-of-concept CL formulation of paclitaxel has demonstrated promising therapeutic value in Phase II clinical trials to treat advanced triple-negative breast cancer (Awada et al. 2014) and is under evaluation in a Phase III clinical trial in patients with locally advanced/metastatic pancreatic cancer (NCT03126435). Several other studies have confirmed targeted drug delivery to tumor vessels utilizing CLs as a successful approach in cancer therapy (Löhr et al. 2012; Lila et al. 2009). However, the use of CLs in the clinic has been challenged by several variable factors, such as increased opsonization and rapid clearance by the MPS or poor tumor penetration, which requires further optimization (Liu et al. 2020).

The microenvironment of tumor tissue shows pathological abnormalities, including hypoxia, acidic pH, high levels of specific enzymes, and altered concentration of adenosine triphosphate (ATP) and redox potential (Maleki et al. 2019). Correspondingly, the overexpression of matrix metalloproteinases (MMPs), including MMP-2 and MMP-9, was shown as principal mediators of tumor invasion and metastasis where decomposition of the extracellular matrix is critical for tumor progression (Piperigkou et al. 2021).

**Scheme 1:**
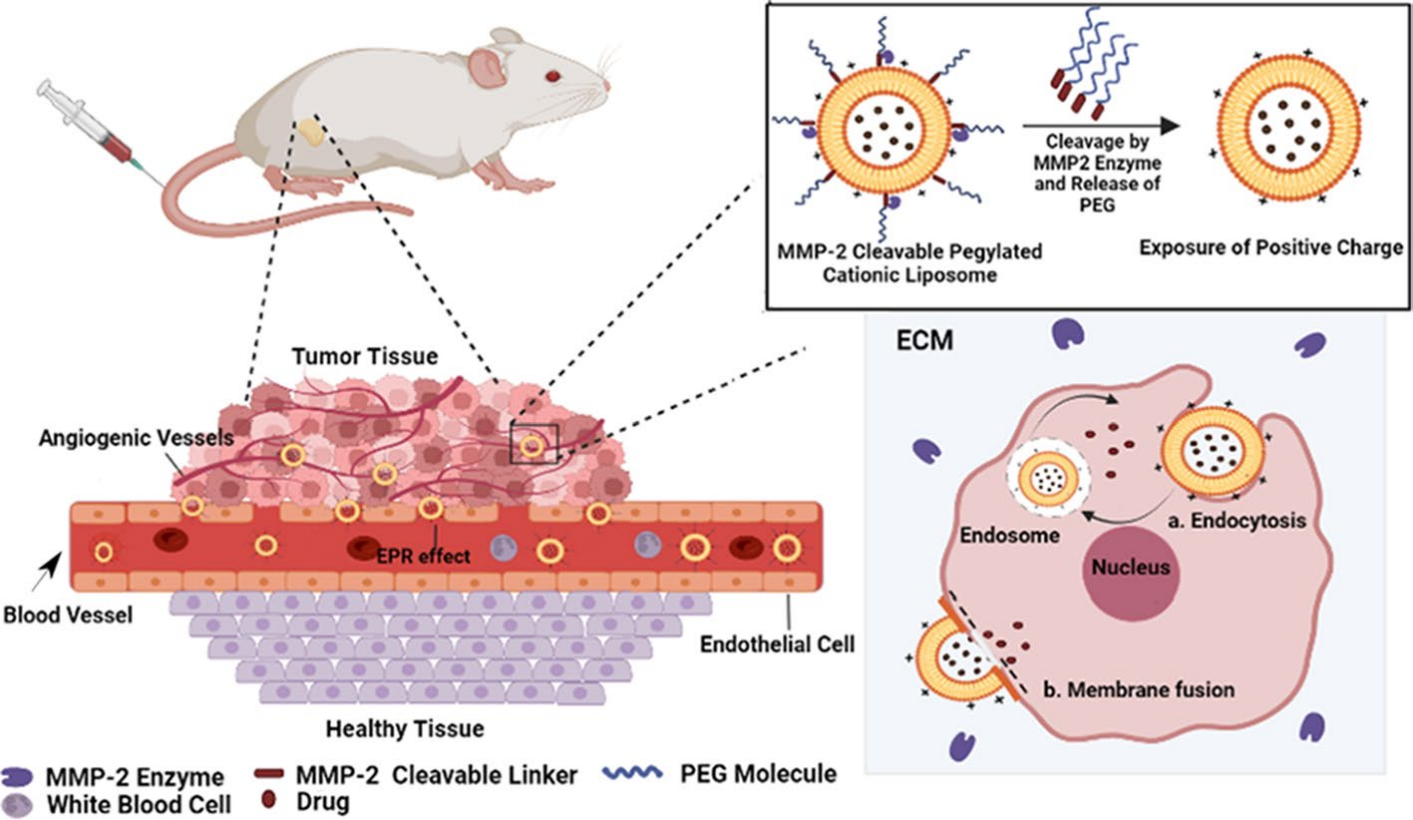
The matrix metalloproteinase-2 (MMP-2) cleavable PEGylated liposomes expose their cationic lipids in response to the upregulated MMP-2 at the tumor microenvironment, resulting in enhanced liposomal uptake and drug delivery to tumor cells and vasculature

Here, we report on the synthesis of MMP-2 cleavable PEGylated CLs, which expose their cationic lipids in response to the upregulated MMP-2 at the tumor microenvironment, resulting in enhanced liposomal uptake and drug delivery to tumor cells and vasculature. To achieve this, the PEG molecules were conjugated to the primary amine groups on the surface of liposomes via a MMP2-cleavable octapeptide linker (Gly-Pro-Leu-Gly-Ile-Ala-Gly-Gln). Activated MMP-2 is reported to cleave this peptide linker at a site between Gly and Ile (Lin Zhu et al. 2012). The design and delivery strategy of these novel liposomal nanocarriers is illustrated in the Scheme 1. The synthesis, functionality, stability, release, uptake, toxicity, and antiangiogenic activity of the PEG-MMP-2 cleavable linker-lipid conjugate and/or MMP2-responsive Dox-loaded CLs were evaluated in vitro. Moreover, the pharmacokinetic parameters of the formulations were evaluated. The biodistribution and antitumor activities were studied in mice bearing the C26 colon carcinoma.

## Results and discussion

### Synthesis and characterization of the mPEG-MMP-2 cleavable peptide-DOPE conjugate

CLs can adsorb plasma proteins after IV administration, the so-called protein corona (PC), which accelerates their elimination from the blood (Liu et al. 2020). Surface-shielding with PEG molecules is the most commonly used strategy to address this issue (Sasayama et al. 2019). Although PEGylation decreases the formation of PC and expands blood circulation, it can hinder cellular uptake (Pozzi et al. 2014). Surface-engineering of liposomes with stimuli-responsive PEG coatings, such as cleavable PEG-lipid conjugates, have been developed to maintain the stealth role in the blood circulation and promote uptake at the target site upon deshielding of PEG molecules in response to the stimulus (Fang et al. 2017; Caiyan Zhao et al. 2016a). Here, we have introduced the synthesis process for the mPEG-MMP-2 cleavable linker-DOPE conjugate that can respond to the overexpressed MMP-2 in the tumor microenvironment. Incorporating this conjugate in the CL formulation can protect the nanocarrier in the blood from the formation of PC but improve tumor cell penetration at the tumor site.

**Scheme 2:**
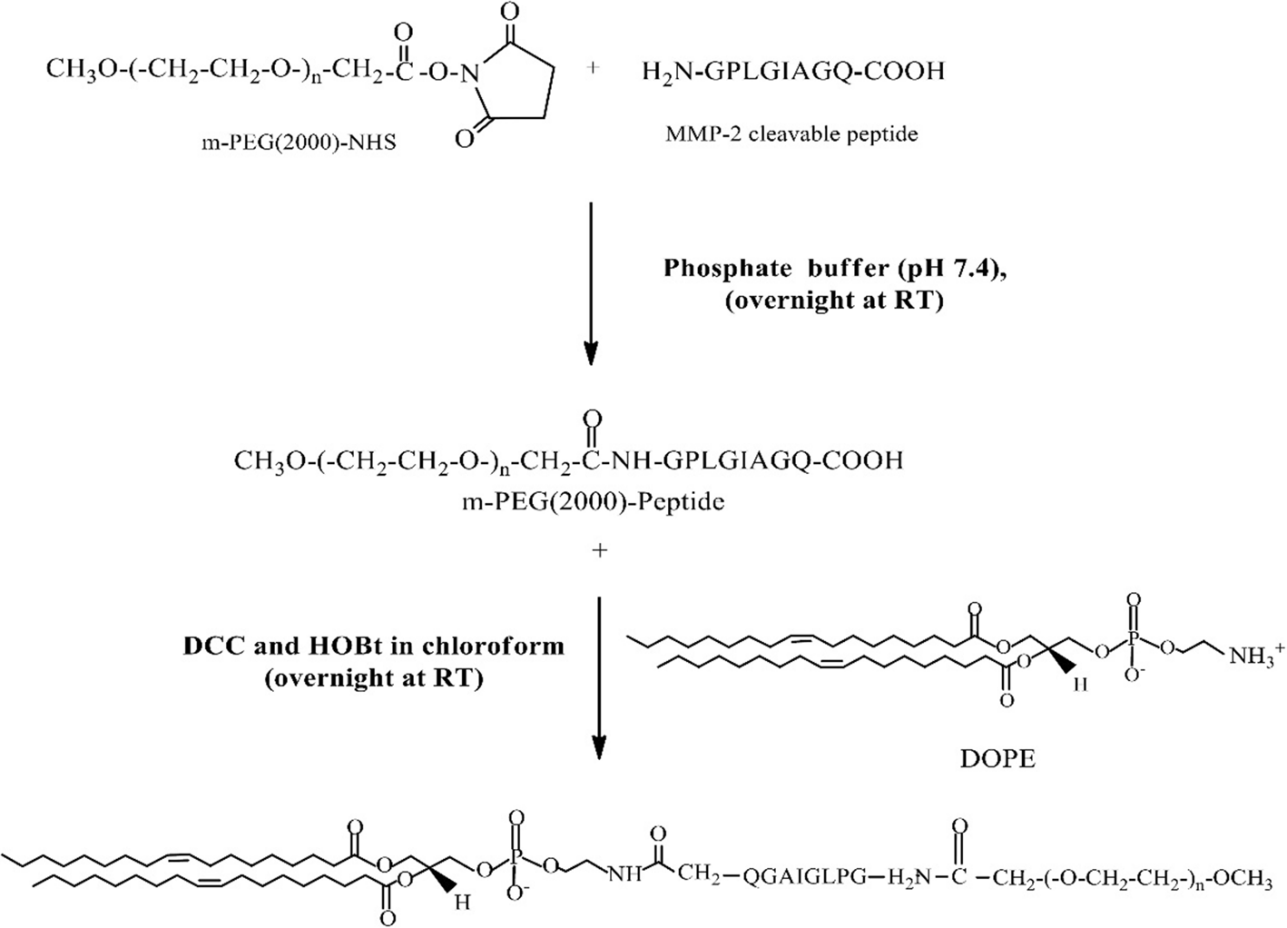
Synthesis of the DOPE-MMP-2 cleavable peptide-PEG conjugate

The MMP-2 cleavable sequence (GPLGIAGQ) was designed based on previous reports (Lin Zhu et al. 2012; Terada et al. 2006). As shown in the Scheme 2, the MMP-2 cleavable peptide was inserted between the PEG polymer and the DOPE lipid in two steps. First, the N-terminus amino group of the peptide was reacted with the NHS ester of the mPEG-NHS polymer (amide linkage) and purified by dialysis. The HPLC chromatograms confirmed the reaction, with a shift in the peak retention times from 3.25 min for the MMP-2 cleavable peptide to 2.60 min for the mPEG-MMP-2 linker-DOPE conjugate (Additional file 1: Fig. S1). The conjugation reaction was further validated by the TLC results as shown in the Additional file 1: Fig. S1. Then, the purified mPEG-peptide conjugate was activated in the presence of DCC/HOBt and conjugated to the DOPE lipid through a condensation reaction (Scheme 2). The PEG-peptide-DOPE conjugate formation was confirmed via TLC followed by iodine staining (Additional file 1: Fig. S2). Moreover, ^1^H NMR spectra in CDCl_3_ indicated that the amino hydrogen of the DOPE lipid at 8.5 ppm moved upfield as a result of the amide bond formation in the conjugate as reported before (Song et al. 2018), confirming the successful synthesis process (Additional file 1: Fig. S3).

### Physicochemical characterization of liposomal formulations

Several liposomal formulations composed of DOTAP, HSPC, DOPE, mPEG2000-DSPE, cholesterol, mPEG-MMP-2 cleavable linker-DOPE conjugate and the antioxidant α-tocopherol were fabricated and characterized in terms of particle size, homogeneity, surface charge, and EE% (Table 1). The total lipid concentration of liposomes was set to 50 mM and various lipid compositions were used to obtain neutral, cationic, or anionic liposomes. Moreover, we compared non-PEGylated liposomes against PEGylated liposomes containing the non-cleavable PEG-lipid or the mPEG-MMP-2 cleavable linker-DOPE conjugate. The F1 formulation containing HSPC, cholesterol, DSPE-mPEG2000, and α-tocopherol at 56.7/38/5.3/0.2 molar ratio, respectively, was selected as its phospholipid composition represents the marketed Caelyx^®^. Since the stealth PEG layer in Caelyx^®^ formulation might restrict its interaction with the cell membrane, we then prepared the F2 and F3 formulations containing the mPEG-MMP-2 cleavable linker-DOPE conjugate at two different molar ratios of 2.5 and 5%, respectively. These formulations were designed to respond to the overexpressed MMP-2 enzyme at the tumor site and release their surface PEG for increased cellular interaction and uptake. To prepare CLs, 10 mol% DOTAP was added to the composition of the F4-F8 formulations.

**Table 1:** Physicochemical characteristics of Dox-loaded liposomal formulations

Formulation	Lipid composition	Particle size (nm ± SD)^a^	PDI^b^ ± SD	Zeta-potential (mV) ± SD	EE%^c^
F1 (Caelyx^®^)	HSPC/Chol/DSPE-PEG2000/α-tocopherol (56.7/38/5.3/0.2)	96.000 ± 2.12	0.10 ± 0.01	− 10.2 ± 0.38	100
F2	HSPC/Chol/DOPE-peptide-m-PEG/α-tocopherol (59.5/38/2.5/0.2)	159.501 ± 3.51	0.19 ± 0.02	− 7.61 ± 0.29	94
F3	HSPC/Chol/DOPE-peptide-m-PEG/α-tocopherol (57/38/5/0.2)	160.712 ± 1.92	0.18 ± 0.03	− 5.31 ± 0.31	90
F4	DOTAP/HSPC/DOPE/Chol/α-tocopherol (10/50/2/38/0.2)	127.173 ± 1.31	0.13 ± 0.01	+ 17.6 ± 0.22	88
F5	DOTAP/HSPC/Chol/DOPE-peptide-PEG/α-tocopherol (10/49.5/38/2.5/0.2)	130.931 ± 2.25	0.12 ± 0.04	+ 14.9 ± 0.13	95
F6	DOTAP/HSPC/Chol/DOPE-peptide-PEG/α-tocopherol (10/47/38/5/0.2)	124.702 ± 4.12	0.15 ± 0.02	+ 10.3 ± 0.41	84
F7	DOTAP/HSPC/DOPE/DSPE-PEG2000/Chol/α-tocopherol (10/47/2.5/2.5/38/0.2)	120.816 ± 3.19	0.10 ± 0.01	+ 13.6 ± 0.16	92
F8	DOTAP/HSPC/DOPE/DSPE-PEG2000/Chol/α-tocopherol (10/42/5/5/38/0.2)	170.312 ± 2.95	0.18 ± 0.04	+ 3.67 ± 0.34	87

^a^Diameter of liposomes (Z-average)

^b^Polydispersity index

^c^Encapsulation efficiency. Values are presented from triplicate measurements of each formulation. Data are presented as mean ± standard deviation (SD)

**Fig. 1:**
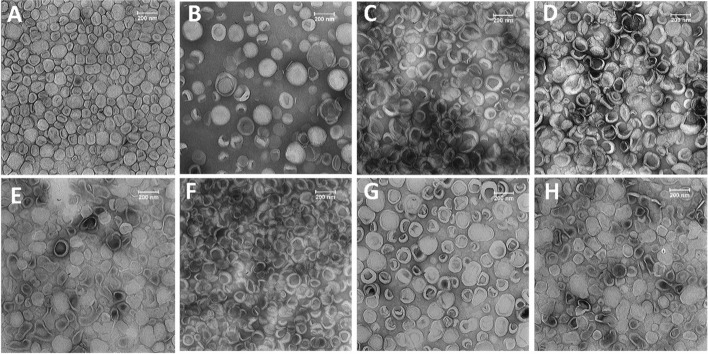
Transmission electron microscopy (TEM) images of the negatively stained liposomes. **A** Caelyx^®^, **B** F2, **C** F3, **D** F4, **E** F5, **F** F6, **G** F7, and **H** F8 formulation

Fabrication of nanoparticles with desirable size, homogeneity, and surface characteristics plays a key role in improving their EE%, stability, drug release rate, cell uptake, and biodistribution (Bahari and Hamishehkar 2016). It is well documented that formulations with a size of ~ 50–200 nm progressively accumulate in the tumor utilizing the EPR effect while showing a reduced uptake profile by the liver and spleen, and limited renal clearance (Ray et al. 2019). The hydrodynamic size of all formulations ranged between 120 to 170 nm with a narrow distribution (PDI ˂ 0.2), and covering a range of surface charges (− 10.2 mV to + 17.6 mV) (Table 1). Incorporating the mPEG-MMP-2 cleavable linker-DOPE conjugate into the composition of liposomes at 2.5% molar ratio of total lipids (F5) caused no significant change in their Z-average and PDI values as compared to their counterpart containing the mPEG-DSPE lipid (F7). Due to the small size of the MMP-2 cleavable peptide (~ 711 Da) with a nearly neutral charge and low molar ratio (2.5%), its incorporation had minimal influence on particle size, PDI, or zeta-potential of the liposomes. Negative staining TEM was also used to assess the morphological characteristics of the formulations (Fig. 1). The TEM image demonstrated the spherical shape of the liposomes with a homogeneous size distribution.

### Release study

**Fig. 2:**
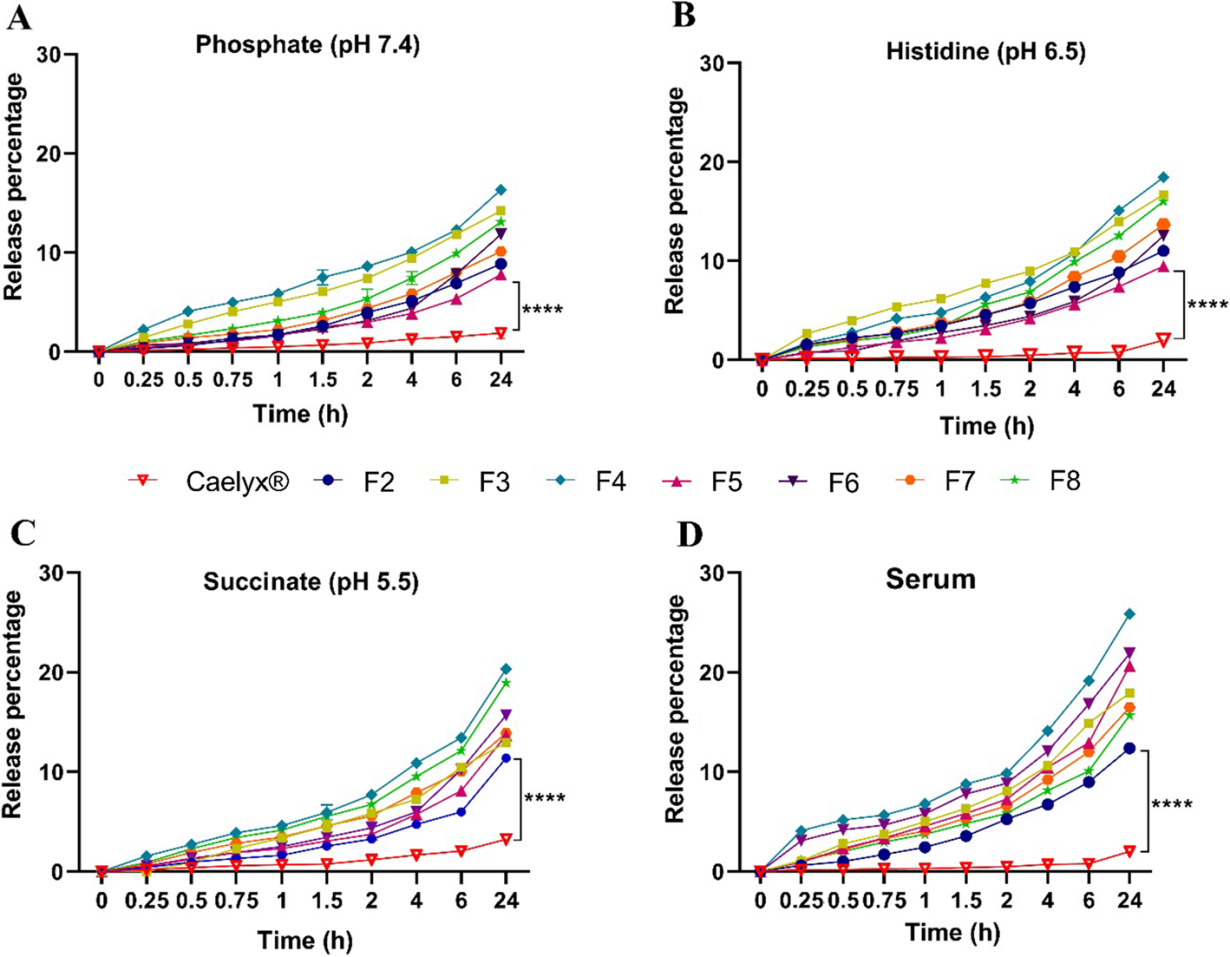
In vitro release profile of Dox from liposomal formulations at pHs of 7.4 (**A**), 6.5 (**B**), 5.5 (**C**), and in serum (**D**) at 37 °C with mild stirring under sterile conditions. The statistical significant difference is determined as follows: ****P < 0.0001. The test was done in triplicate and data are shown as mean ± SD (*n* = 3)

Liposomes release profiles can influence their pharmacokinetics and the required dosage, which in turn affects drug effectiveness. (Yang Zhao et al. 2016b). In this study, we assessed the release pattern of the liposomal formulations at three different pHs (pH 7.4, physiological fluids; pH 6.5, tumor microenvironment; and pH 5.5, late endosomes) over 24 h. As shown in Fig. 2, all formulations had an increased release profile as compared to the Caelyx^®^. This was postulated as a result of incorporation of unsaturated lipids such as DOTAP and DOPE with kinked tails and looser alignment in their formulation in contrast to the saturated lipids in the formulation of Caelyx^®^ which align in tight packings (Olsman et al. 2020). Moreover, Dox release from liposomes had the lowest rate at pH 7.4 compared to acidic pHs perhaps due to the destabilization of lipid carriers at lower pH as reported previously (Park et al. 2014). At lower pH, the van der Waals interactions between phospholipids reduce, consequently changing membrane structure and increasing drug release (Karimi et al. 2020). On the other hand, protonation of choline groups may cause electrostatic repulsion, resulting in the instability of liposomal formulations. Additionally, DOPE is an unsaturated phospholipid that exhibits conformational changes with pH changes. In acidic pH, the structure of DOPE can be transformed to an inverted hexagonal (H_II_), thus disrupting liposome bilayer (Mochizuki et al. 2013). Interestingly, Dox release from the non-PEGylated liposome (F4) had the highest rate as compared to other formulations at pH 7.4. This behavior could be attributed to the steric hindrance of the PEG polymer chains that interfere with drug release from nanocarriers (Haghiralsadat et al. 2018).

The interaction of liposomes with serum components is another factor that affects their stability and drug release. For this purpose, release studies were also carried out at the physiologic pH of 7.4 and in the presence of FBS. As shown in Fig. 2D, 13–30% of the total amount of the encapsulated Dox was released from CLs after 24 h incubation in dextrose/FBS buffer, which was significantly higher (*p* < 0.0001) than Caelyx^®^. These results might be attributed to the higher interaction and lipid transfer from CLs to negatively charged serum albumin and lipoproteins, thus destabilizing the liposome membrane integrity or fusion/aggregation which results in accelerated drug release (Sharifi et al. 2020). However, the cumulative release of Dox from CL formulations reached to ~ 30% in dextrose/FBS buffer over 24 h, indicating their acceptable stability during blood circulation.

### Physicochemical stability

Physicochemical stability during the storage period determines the final product's long-term stability and shelf-life. The physicochemical properties of liposomes and their microenvironment can influence drug leakage, aggregation, fusion, precipitation, and hydrolytic or oxidative degradation of the lipid membranes (Nakhaei et al. 2021). Here, the stability of liposomes was monitored under hypothermic storage conditions (2–8 °C) over 8 months in size, zeta-potential, appearance, homogeneity, and EE%. As illustrated in Additional file 1: Table S1, the F1, F4, F5, F6, and F7 liposomal formulations were approximately stable without any considerable changes in size, PDI, surface charge, and EE%. In contrast, a moderate increase in size was detected for F2, F3, and F8 formulations which may be due to increased drug leakage from these liposomes and their lower magnitude of surface charge. Moreover, visual inspection affirmed no changes in the appearance of any formulation and no sedimentation during the storage period. The electrostatic repulsion can overcome the aggregation tendency between colloidal particles and impart long-term stability (Sultana et al. 2020). Besides, steric stabilization using PEG coating creates a repulsive force, minimizing particles interaction or aggregation (Sopyan and Gozali 2020). The incorporation of HSPC in this study as the main lipid with a high phase transition temperature (Tm: 55 °C, (Li et al. 2015)) resulted in more stable liposomal formulations. Additionally, cholesterol has impacted liposome stability with phospholipid molecule packing, decreased bilayer permeability, increased bilayer rigidity, and reduced drug leakage (Karimi et al. 2020). The use of α-tocopherol as an antioxidant in the composition of liposomes, the low temperature of storage, and buffering medium at pH 6.5 in which phospholipids have shown the lowest rate of hydrolysis can also affect the chemophysical stability of liposomal formulations during storage (Mirafzali et al. 2014).

### Cell binding and uptake

**Fig. 3:**
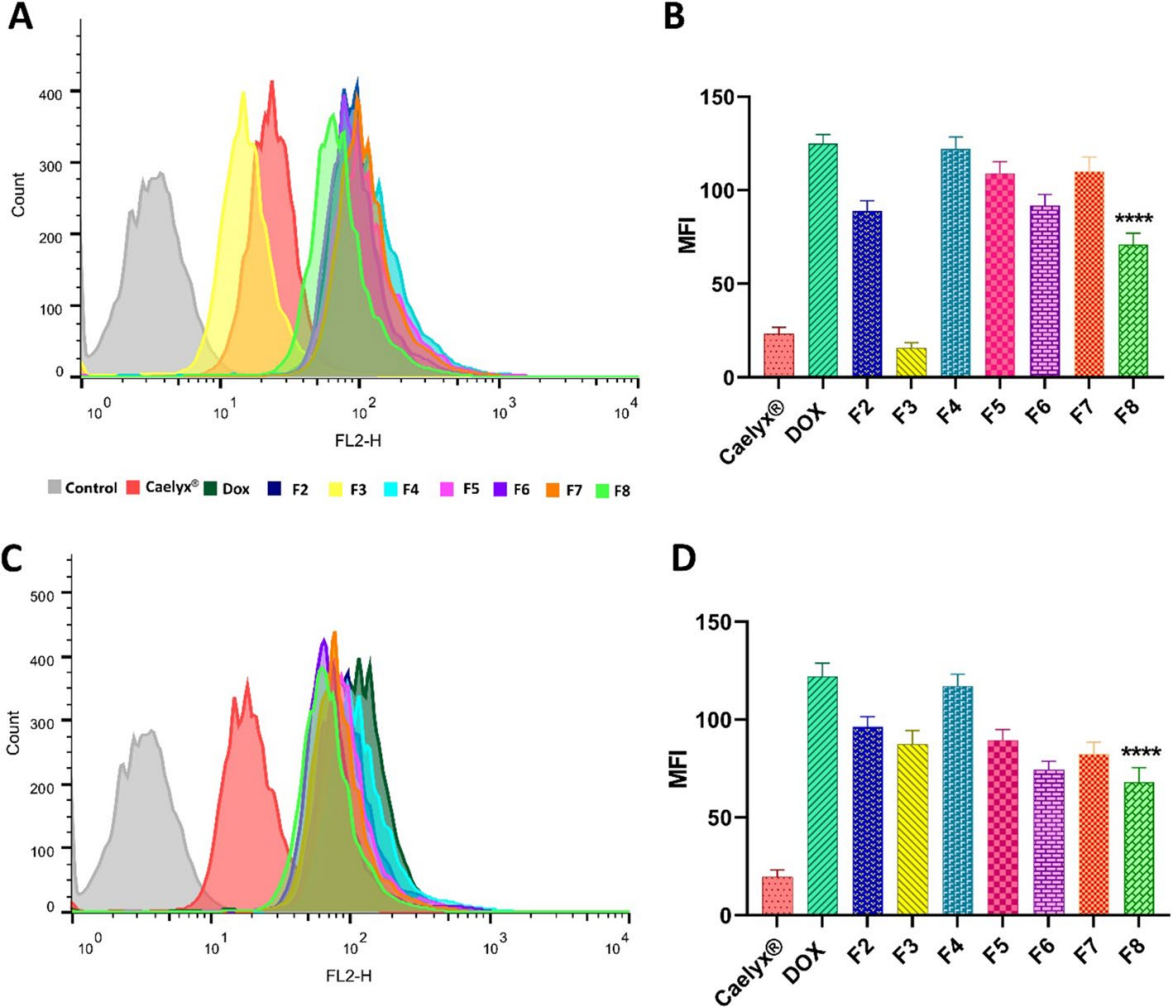
Cellular interaction (**A**, **B**) at 2–8 °C and cell uptake (**C**, **D**) at 37 °C of the free Dox and Dox-loaded liposomal formulations in C26 tumor cells. Untreated cells were applied as the control group. Results were assessed by flow cytometry and expressed as MFI. The results are shown as means ± SD (*n* = 3 independent experiments). Statistical significance is compared to the Caelyx^®^ and designated as follows: ****P < 0.0001. The test was performed in triplicate

Achieving the best therapeutic response is dependent on efficient cellular uptake of the encapsulated drug. The size and charge of liposomes determine the level of cell–liposome interactions, which consequently affect the cellular uptake (Olsman et al. 2020). Liposomes are internalized into cells in a temperature-dependent manner and endocytosis in low temperature is negligible. The cellular binding and uptake of liposomal formulations were tested on C26 cells at 4 °C and 37 °C by flow cytometry and the results are depicted in Fig. 3. The non-PEGylated F4 CLs showed the highest rate of cell interaction and uptake in comparison with other liposomal formulations which is consistent with the results of prior studies (Niu et al. 2010). These results confirmed that the positive zeta-potential of CLs induces greater electrostatic interaction with cells and thereby induces internalization via endocytic pathways (Dass 2003). It is also important to consider degree of PEGylation as a steric barrier in comparisons between different formulations. In general, the liposomal formulations having a 2.5% molar ratio of the PEG-Lipid showed higher cellular interaction than their counterparts having a 5% molar ratio of the PEG-lipid (F2 vs F3 (*P* < 0.0001); F5 vs F6 (*P* < 0.05); and F7 vs F8 (*P* < 0.01)). Also, cellular uptake was higher in F2, F5, and F7 formulations than their counterparts but this was not statistically significant. Nevertheless, it has been shown that partial PEGylation of CLs does not interfere with their affinity to attach cell surfaces (Campbell et al. 2009). Despite a slightly negative surface charge of the F2 and F3 formulations, these liposomes showed uptake rates comparable to the more positively charged formulations (F5 and F6). It should be noted that all these formulations were developed with the cleavable-PEG-lipids and deshielding of the PEG-coating from their surface over the incubation time could have resulted in their fusion with cellular membrane and uptake in vitro. In the case of Caelyx^®^, the low cellular interaction and uptake can be attributed to its negative surface charge (~ − 15 mV) which leads to electrostatic cell-surface repulsion (Jung et al. 2009). Moreover, it should be noted that Dox release over 3 h time interval of the cell binding and uptake studies might have played a minimal effect on the detected MFI as the maximum release rate of the liposomal formulations was only limited to ~ 10% total amount of encapsulated Dox over this time. Besides, there was a clear difference in cell interaction (*P* < 0.0001) and uptake (*P* < 0.001) between F3 and F4 formulations which had similar release profiles.

**Fig. 4:**
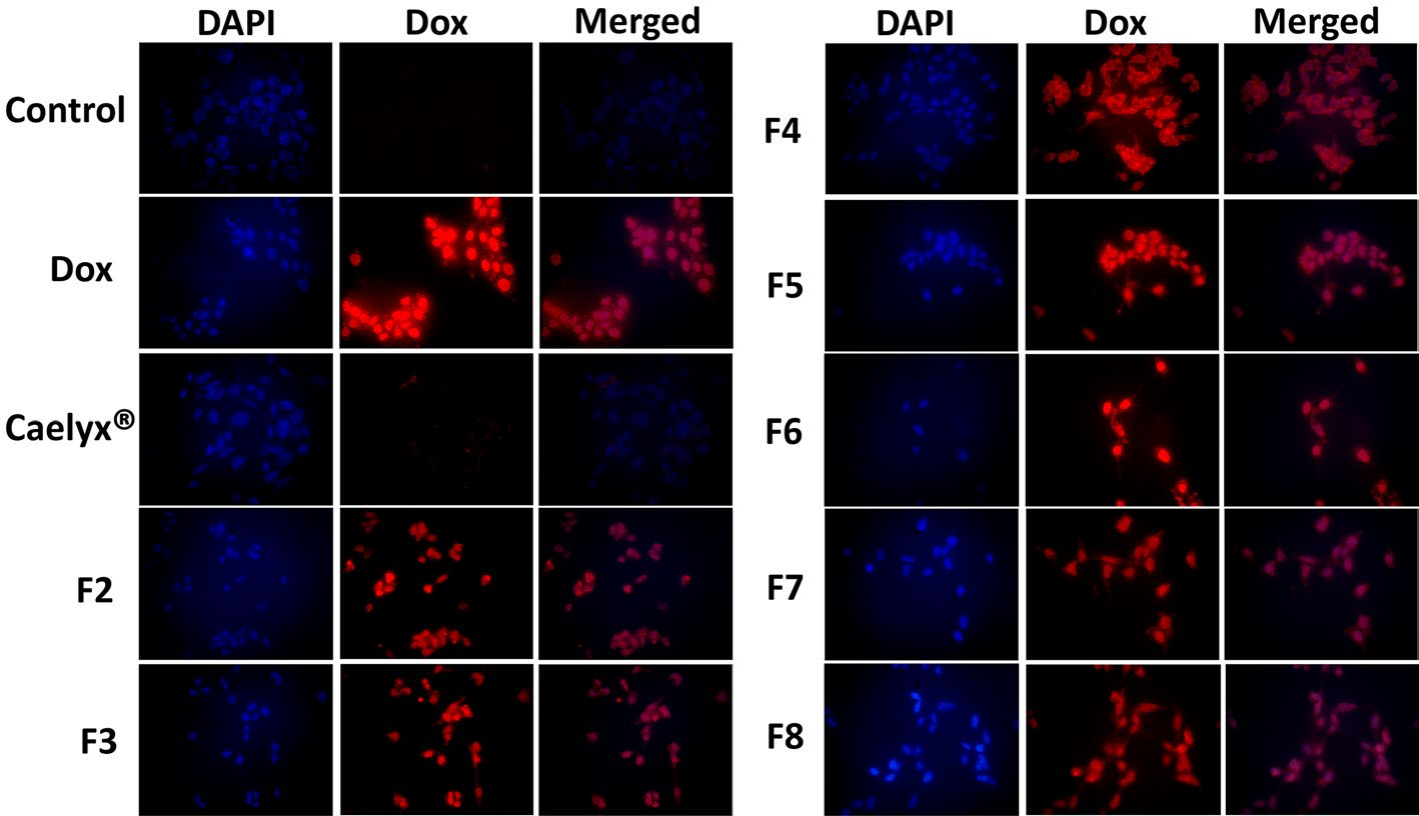
Fluorescence microscopy of C26 cancer cells treated with the free Dox (10 µg/mL) or Dox-loaded liposomal formulations after 3 h incubation at 37 °C

We further evaluated intracellular Dox after treatment with different formulations using fluorescence microscopy (Fig. 4). Similar to the flow cytometry results, the stronger fluorescence intensity was observed following treatment with the free Dox consistent with the results of previous studies (Wenxi Wang et al. 2017). As expected, CLs internalization was remarkably higher than the Caelyx^®^. These results further support our hypothesis that positive zeta-potential of CLs mediates electrostatic interaction with the negatively charged tumor cell surface.

### MMP-2 expression

**Fig. 5:**
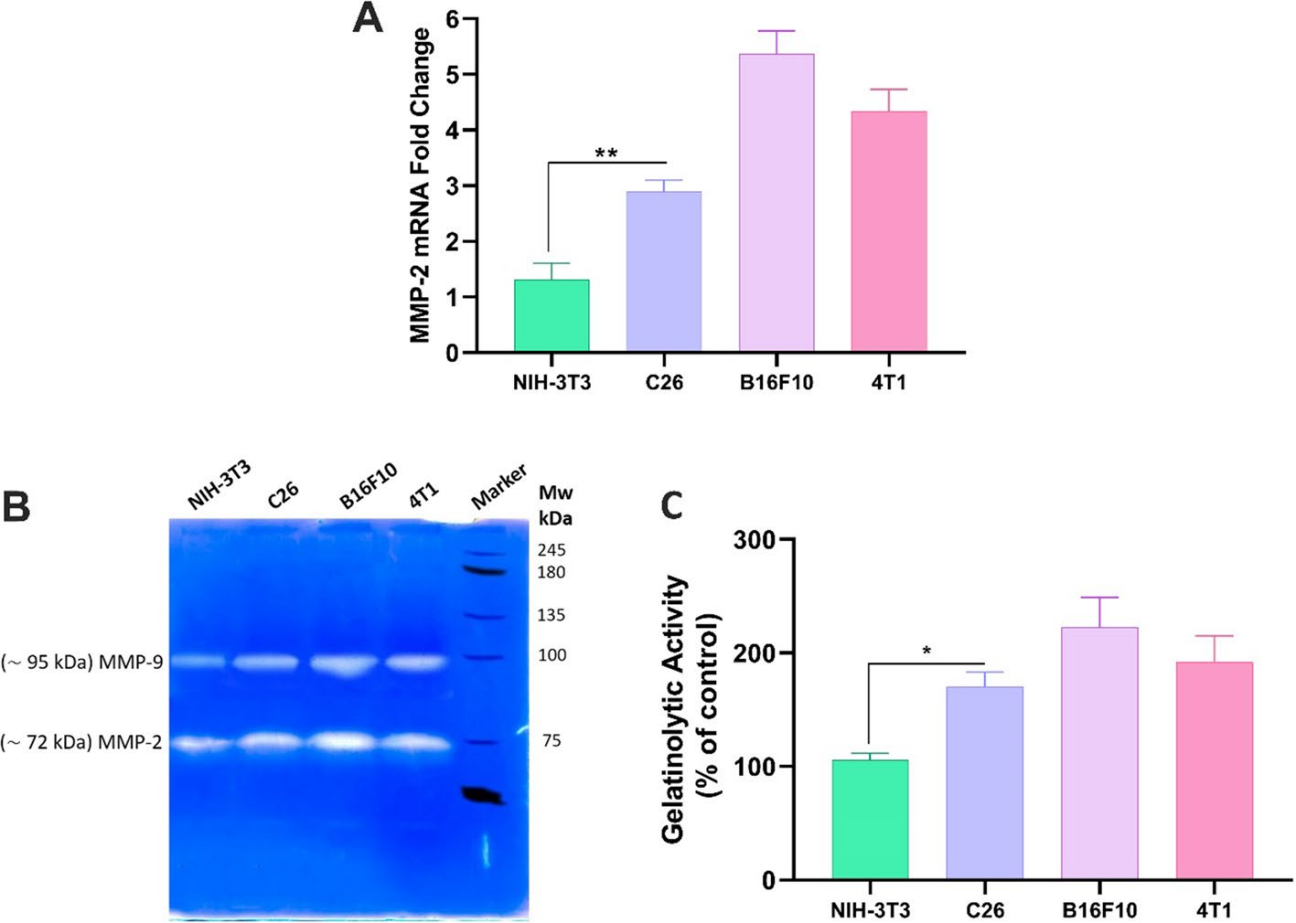
MMP-2 mRNA expression in the NIH-3T3, C26, 4T1, and B16F10 cells (**A**). Gelatinase zymogram (**B**) and densitometry analysis (**C**) of MMP-2 secretion in NIH-3T3, C26, 4T1, and B16F10 cells. All experiments were performed in triplicates and results are presented as the mean ± SD. Statistical significance is compared to the control group and shown as follows: *P < 0.05, **P < 0.01

mRNA expression and activity of MMP-2 in the normal NIH-3T3 or C26, 4T1, and B16F10 metastatic cell lines were evaluated using qRT-PCR and gelatin zymography, respectively (Fig. 5). The zymogram of the metastatic cells showed considerably higher MMP-2 expression as compared to the normal NIH-3T3 cells (Fig. 5b, c). Moreover, the results of the qRT-PCR analysis demonstrated substantially (*P* < 0.05) higher MMP-2 mRNA expression of the metastatic cell lines as compared to the normal NIH-3T3 cells (Fig. 5a).

### In vitro anticancer effects of Dox formulations

**Fig. 6:**
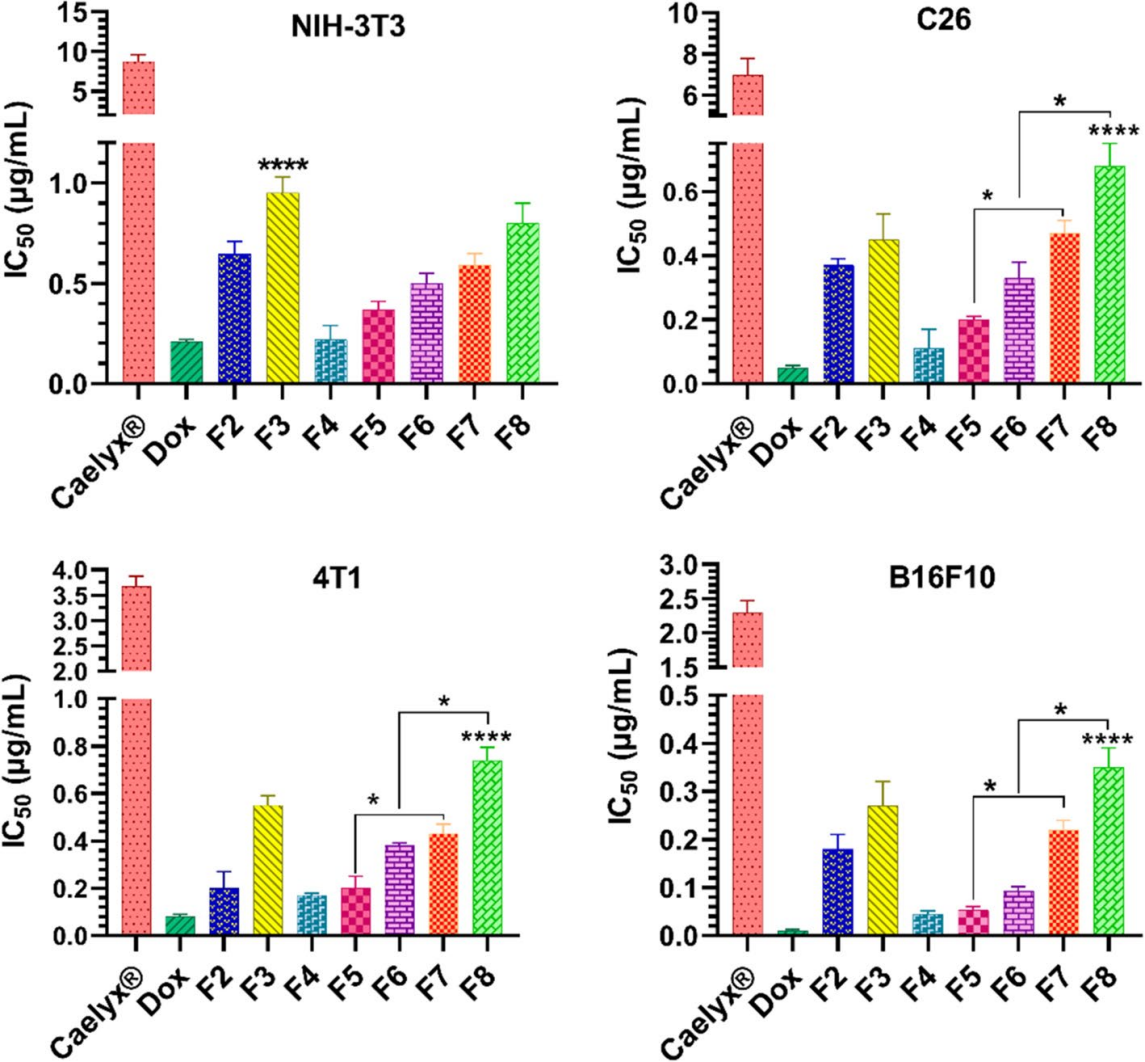
IC_50_ values of the free Dox and Dox-loaded liposomes in the NIH-3T3, C26, 4T1, and B16F10 cells. Statistical significance is compared to the Caelyx^®^ and shown as follows ****P < 0.0001. The data are provided as mean ± SD (*n* = 3)

The cytotoxicity of the free Dox and Dox liposomal formulations were measured in vitro against the normal NIH-3T3 cells and the C26, 4T1, or B16F10 metastatic cells using MTT assay (Fig. 6). All formulations showed dose-dependent cytotoxicity against all cell lines. Free Dox showed lower IC_50_ values compared to the Dox liposomal formulations, which is due to efficient uptake of Dox by tumor cells (Hwang et al. 2007). As shown in Fig. 6, all formulations exhibited higher cytotoxicity against metastatic cell lines than the normal NIH-3T3 cells. Also, all Dox-loaded liposomal formulations had significantly higher cytotoxicity (*P* < 0.05) in comparison with the commercial Caelyx^®^. This could be attributed to the lower cellular interaction and uptake of Caelyx^®^ relative to the CLs as shown in the results of the cell binding and uptake. As regards, the non-PEGylated CL (F4) formulation of Dox demonstrated the lowest IC_50_ value among others, which is probably due to its high cellular uptake as shown before. Although F2 and F3 formulations were classified as nearly neutral liposomes, they showed lower IC_50_ values relative to Caelyx^®^ which could be as a result of higher Dox release rates than Caelyx^®^ as shown in the release results. Interestingly, the CLs containing mPEG-MMP-2 cleavable peptide-DOPE conjugate (F5 and F6) showed statistically more potent anticancer activity (*P* < 0.05) than their non-cleavable PEGylated counterparts (F7 and F8) in the C26, 4T1, and B16F10 metastatic cell lines. In C26 and 4T1 cell lines, the potency of F5 and F6 liposomes was roughly ~ 2- to 2.35-fold more than the F7 and F8 liposomes. In B16F10 cell line, an enhanced potency up to fourfold was observed. Therefore, it can be postulated that the mPEG-peptide-DOPE degradation in the presence of the secreted MMP-2 enzyme in the conditioned medium causes enhanced electrostatic interaction and cellular uptake of the deshielded CLs.

### Antiangiogenic activity

**Fig. 7:**
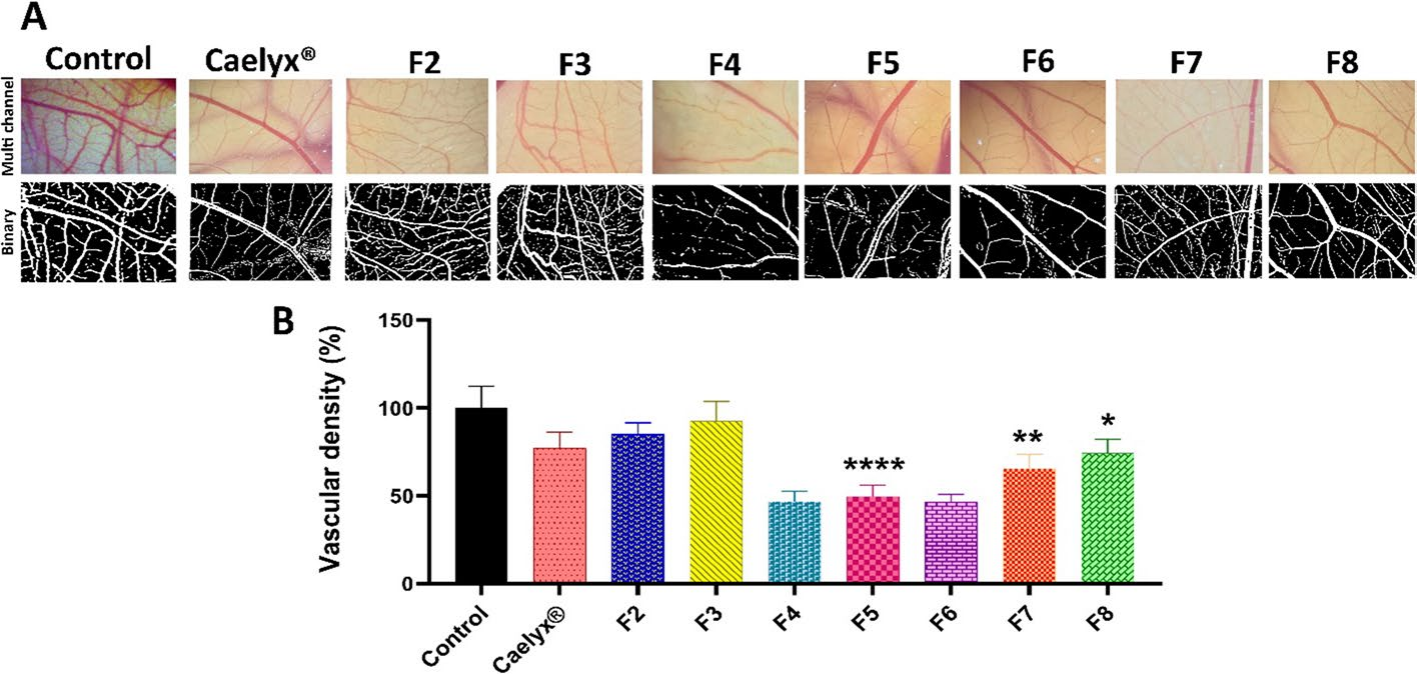
CAM vasculature development on day 12 of egg development treated with liposomal formulations. (**A**) shows the stereomicroscope of the CAM vessels. (**B**) shows relative vascular density as compared to the control group. Data are expressed as mean ± standard deviation (*n* = 3). The statistical significant differences are designated as follows: *P < 0.05, **P < 0.01, ****P < 0.0001

CLs have the ability to target endothelial cells in tumors (Dicheva et al. 2013; Abu-Lila et al. 2009), hence their antiangiogenic activity was further investigated in this study. Among the in vivo models to monitor tumor angiogenesis, CAM assay offers a simple, cheap, and less sentient alternative for animal research. CAM is a highly vascularized network that is created by the fusion of the chorion and allantois membranes within 4–5 incubation days of the chick embryo. CAM has crucial role during embryonic development, including calcium transport from the egg shell, gas exchange, ion and water reabsorption from the allantoic fluid and acid–base hemostasis in the embryo. CAM becomes readily accessible on day 8 of incubation with rapid angiogenesis from day 8–11, which provides great opportunity to test therapeutic agents (Nik et al. 2019; Ribatti et al. 2020). Figure 7 demonstrates the image of CAM vessels on day twelve of egg embryo development (72 h post-treatment). Normal angiogenesis with consistent directional patterns was found in the CAM of the control group. The CAM vasculature in CL-treated eggs became less dense, with an uneven pattern or even blind-ended.

The quantitative analysis results of the angiogenesis in the chicken CAM are shown in Fig. 7. The CL-treated groups had were significantly reduced number of nodes and branches compared to the non-treated control group, indicating their potential to prevent tumor angiogenesis.

### Pharmacokinetic study

The pharmacokinetic parameters of the free Dox and Dox-loaded liposomes were investigated post-i.v. administration of an equivalent dose of 10 mg/kg of Dox in healthy BALB/c mice (Table 2; Additional file 1: Table S2). The results demonstrated an increased half-life of Dox (~ 3.62 h) when encapsulated into liposomes (~ 20 h). The analysis of Dox exposure in the blood as summarized by the area under the first moment curve (AUMC) when encapsulated in neutral liposomes (Caelyx^®^ and F2) was approximately 4 times higher than those of Dox-loaded DOTAP cationic liposomes (F4). This formulation with the highest magnitude of cationic charge showed highest clearance rate and *V*_ss_ among other liposomes, indicating its rapid elimination and tissue uptake. Macrophages in the liver and spleen and endothelial cells in capillaries of the lung and anterior pituitary were reported to avidly bind and take up CLs (Thurston et al. 1998). On the other hand, the CLs containing the DOPE-peptide-PEG conjugate in their structure (F5 and F6) showed comparable half-life, Cl, and AUMC as of the commercial Caelyx^®^. Surface coating with the hydrophilic PEG polymer has widely been utilized to reduce non-specific adhesion of liposomes to plasma proteins, MPS uptake, or recognition by immune cells, leading to prolonged circulation time in vivo.

**Table 2:** Pharmacokinetic parameters of the free Dox and Dox-loaded liposomal formulations

Treatment group	MRT (h)	AUMC (µg*h^2^/mL)	AUC (µg*h/mL)	Cl (mg)/(µg/mL)/h	V_ss_ (mg)/(µg/mL)	t_1/2_ (h)	K (1/h)
Dox	3.98	28.7	7.2	1.39	7.25	3.62	0.19
Caelyx^®^	15.87	23,089.1	1454	0.007	0.11	12.18	0.056
F4	11.73	2710.6	231	0.043	0.51	9.71	0.071
F5	22.66	18,809.7	830	0.012	0.27	16.42	0.042
F6	27.52	17,748.5	645	0.012	0.42	19.18	0.036

F4: DOTAP/HSPC/DOPE/Chol/α-tocopherol (10/50/2/38/0.2), F5: DOTAP/HSPC/Chol/DOPE-peptide-PEG/α-tocopherol (10/49.5/38/2.5/0.2), F6: DOTAP/HSPC/Chol/ DOPE-peptide-PEG/α-tocopherol (10/47/38/5/0.2)

### Biodistribution study

**Fig. 8:**
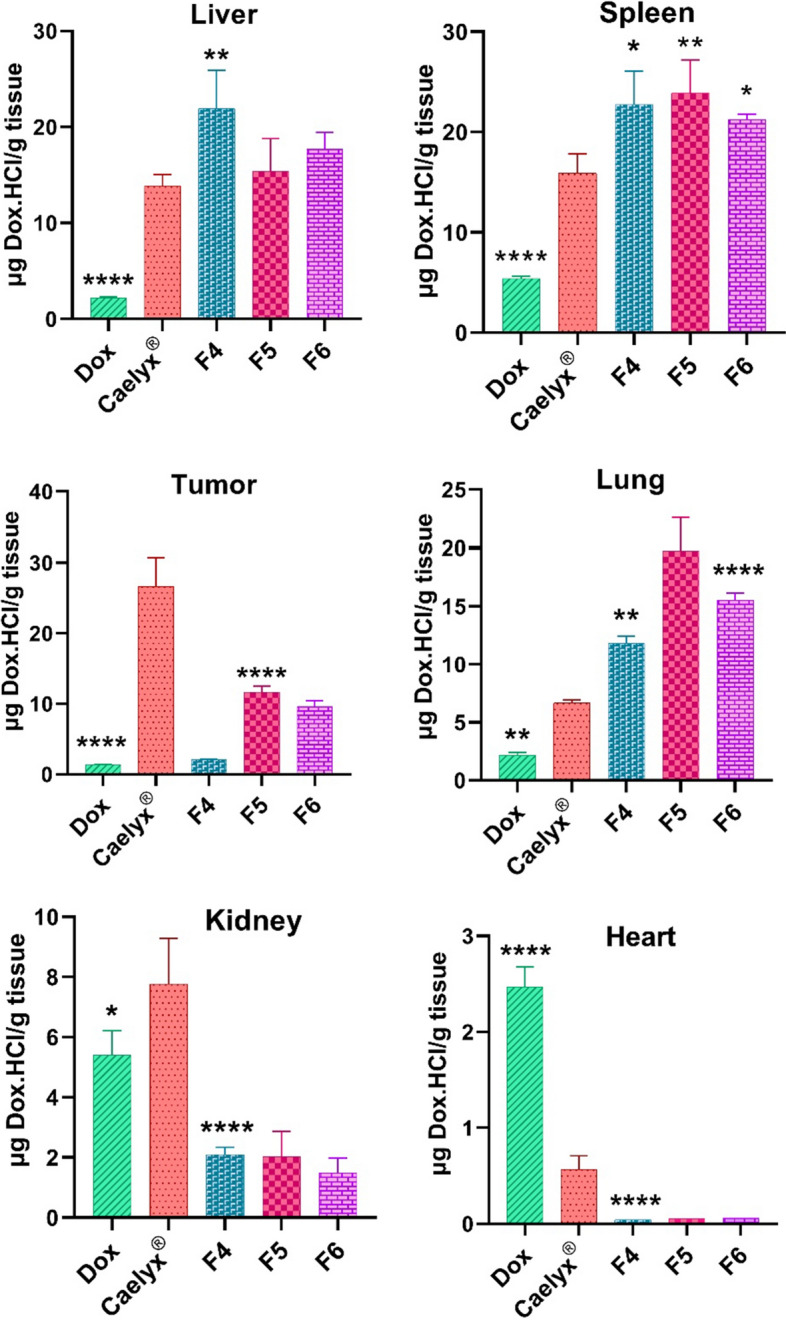
Biodistribution of the free Dox and Dox-loaded liposomes after 24 h. Statistical significances are compared to the Caelyx^®^ and designated as follows: *P < 0.05, **P < 0.01, and ****P < 0.0001

Biodistribution of the free Dox, Caelyx^®^, and Dox-loaded liposomes was evaluated in the main organs including liver, spleen, heart, kidney, lung, and the tumor site in BALB/c mice bearing C26 tumors at 24 h post-injection. As demonstrated in Fig. 8; Additional file 1: Fig. S4, all liposomal formulations of Dox showed significantly (*P* < 0.05) higher tumor accumulation in comparison to the free Dox. As compared to Caelyx^®^, the accumulation of CLs containing the MMP-2 cleavable DOPE-peptide-PEG conjugate (F5 and F6) was significantly (*P* < 0.0001) reduced in the kidney and heart while it was considerably more in the lung. This finding is consistent with earlier studies in which CLs exhibited substantial binding and accumulation in the pulmonary capillary bed as well as uptake by the macrophages in the liver and spleen (Hattori et al. 2021; Samuelsson et al. 2017). Thus, these CL formulations might have promising potential for treating lung malignancies in future.

Caelyx^®^ showed highest tumor accumulation among other formulations. This could be ascribed to its negative surface charge (~ -15.5 mV) and smaller hydrodynamic size (~ 84.3 nm), which lead to low Cl and long circulation in the blood with a higher chance of tumor buildup via the EPR effect (Wibroe et al. 2016). Yet, both F5 and F6 formulations showed significantly (*P* < 0.05) higher Dox concentration at the tumor site in contrast to other Dox-CL formulations (F4, F7 and F8). This enhanced biodistribution pattern of the MMP-2 cleavable Dox-CLs as opposed to the Dox-CLs could be attributed to the effect of PEGyalation and tumor accumulation via the EPR effect, where gradual cleavage of the stealth PEG polymers at the tumor microenvironment increase their binding and uptake over time at the tumor site. It should be noted that despite PEGylation, a fraction of PEGylated-CLs may interact non-specifically with the anionic components in the blood, thus exhibit reduced passive tumor targeting after injection (Wei Zhao et al. 2011).

### Antitumor efficacy and survival analysis

**Fig. 9:**
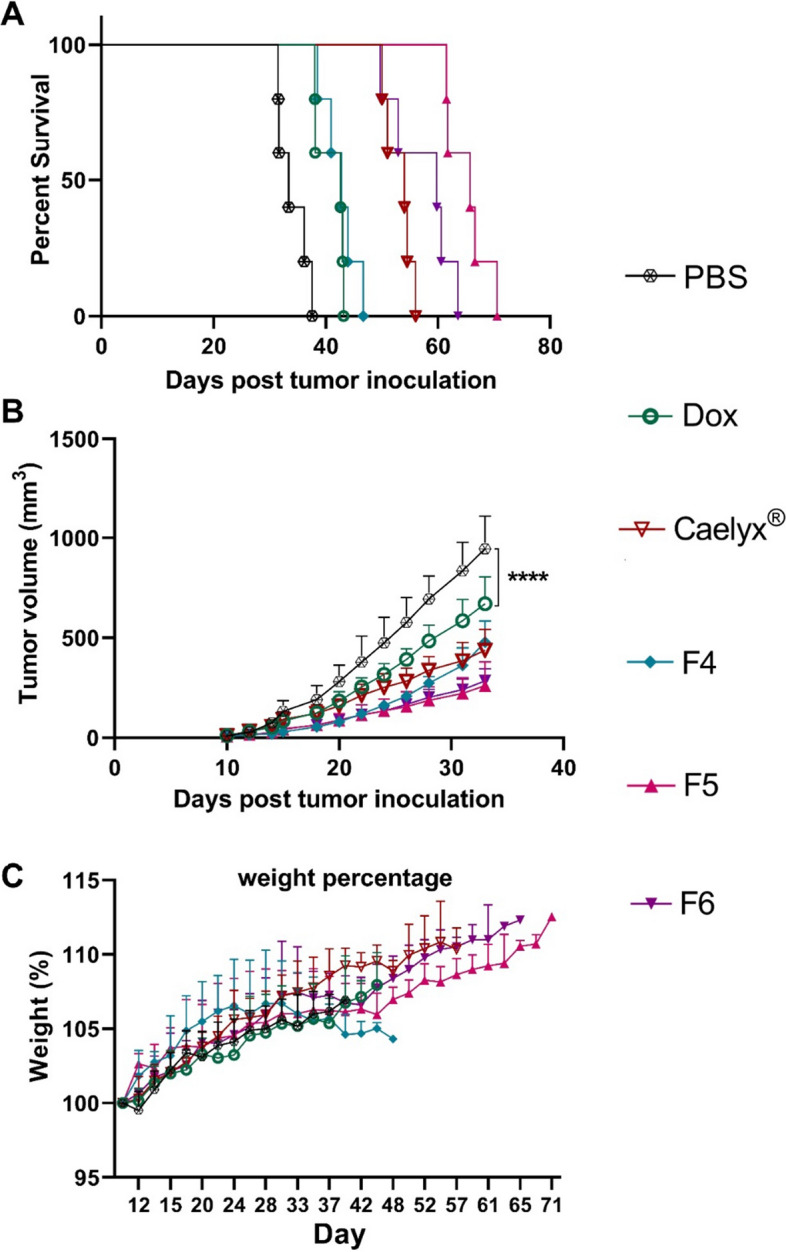
Therapeutic efficacy of the free Dox, Caelyx^®^, and Dox-loaded liposomes in male BALB/c mice bearing C26 tumor. **A** Survival curve, **B** tumor volume and **C** body weight. ****P < 0.0001

The therapeutic activities of the free Dox and liposomes were analyzed against control groups (PBS and control liposomes) in the BALB/c mice bearing C26 tumor. The survival rates and changes in the tumor volume and body weight in response to different treatments are shown over time in Fig. 9A–C and Additional file 1: Fig. S4. It was observed that all liposomal formulations of Dox could decrease the tumor growth rate to a higher extent compared to the free Dox in mice at the same dose. Besides, Dox-loaded PEGylated liposomes showed higher efficacy, irrespective of their surface charge, as compared to the non-PEGylated counterpart (F4). In complement to the previous observations, the survival analysis (up to 70 days) as illustrated in the Kaplan–Meier plot (Fig. 9A) showed that treatment with Caelyx^®^ and Dox liposomal formulations extended animals survival relative to the free Dox and PBS. Notably, mice treated with the MMP-2 cleavable Dox-CLs (F5 and F6) exhibited enhanced survival benefit and tumor growth suppression in comparison with the Caelyx^®^ and free Dox. Additionally, all the liposomal formulations of Dox had no considerable effect on body weight of animals post-treatment which confirms their safe profile in vivo (Fig. 9C). The tumor volume of mice treated with Dox-loaded liposomal formulations was compared to PBS group until 33 days (Fig. 9B). The results indicated that all liposomal formulations reduced significantly (*P* < 0.0001) the tumor volume as compared to the PBS group. Supplementary Fig. S5 shows tumor volume changes of each liposomal formulation separately during 70 days.

The parameters of antitumor study including the MST, TTE, TGD%, and the percentage increased life span (ILS%) are represented in Table 3 and Additional file 1: Table S3. Among Dox-loaded liposomal formulations, the longest MST, and highest TGD% and ILS% were seen in mice treated with the MMP-2 cleavable CLs (F5 and F6). These results could be attributed to the enhanced stability of these formulations in the blood circulation and effective extravasation and tumor cell uptake after PEG-deshielding in response to the overexpressed MMP-2 enzyme at the tumor site as compared to the non-PEGylated CLs or the non-cleavable PEGylated CLs.

**Table 3:** Antitumor effects of the free Dox and Dox-loaded liposomal formulations

Formulation	Time to reach the end point (days)	Tumor growth delay (TGD%)	Median survival time (days)	Percentage increased life span (ILS%)
PBS	34.1 ± 2.7	–	33.4	–
Dox	41.0 ± 2.7	20.96	42.7	27.76
Caelyx^®^	53.9 ± 2.1	58.19	54.0	61.72
F4	42.6 ± 3.1	24.95	42.8	28.18
F5	65.2 ± 3.7	91.48	65.7	96.76
F6	57.3 ± 5.8	68.23	59.8	79.12

### Histological evaluations

**Fig. 10:**
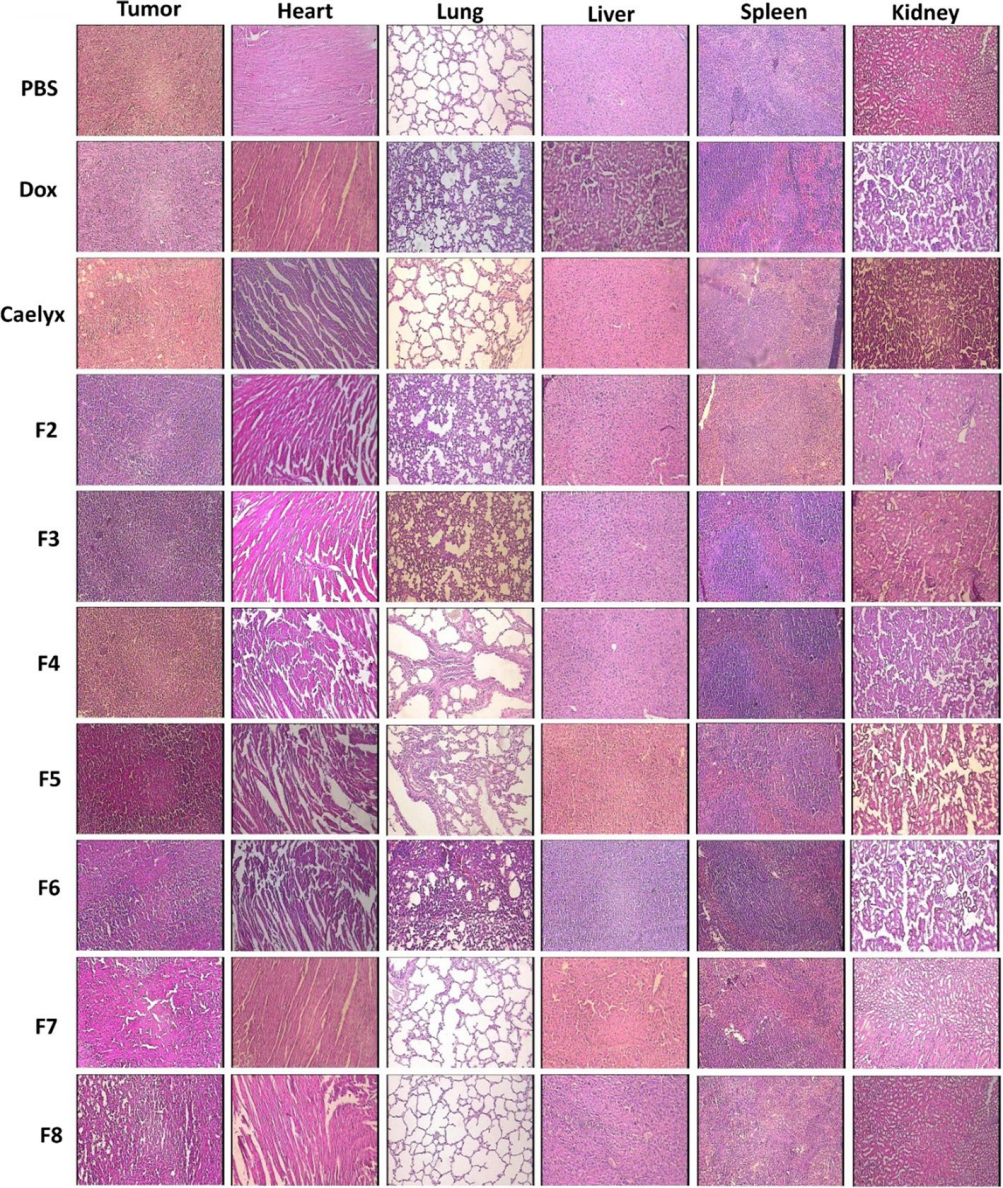
Histological assessment of the main organs in animals treated with the free Dox or Dox liposomal formulations was done via H&E staining of the tumor, heart, liver, kidney, spleen and lung. The evaluation was done on day 20 after i.v. administration of the formulations. The images were presented by 100 × magnification field

The histological analyses were carried out using H&E staining on the heart, lung, liver, spleen, kidney, and tumor to determine the safety of the formulations (Fig. 10). The findings showed that the majority of liposomes had no detrimental effects on the organs. However, the non-PEGylated CL (F4) showed pulmonary inflammation and thickening of the alveolar wall as described elsewhere (Yanyan Zhu et al. 2019). Also, structural lesions induced by cardiotoxic nature of Dox were identified in mice administered with the free Dox, which were in line with those of other investigations (Lei Wang et al. 2016). Besides, the histological sections of the tumors showed that CL formulations caused enhanced necrotic and apoptotic areas at the tumor site as reported before (Ma et al. 2016).

## Conclusions and future perspectives

In the present study, various liposomal formulations of Dox were prepared including neutral, negatively charged and positively charged liposomes with a focus on PEGylated CLs that can release their stealth PEG layer in response to the overexpressed MMP-2 at the tumor microenvironment, and consequently show enhanced tumor cell uptake and antiangiogenic activity. The Dox-loaded MMP-2 cleavable PEGylated CLs showed enhanced efficacy in comparison with the free Dox and liposomes in terms of cytotoxicity, cellular uptake and therapeutic response both in vitro and in the murine model of colon carcinoma. Additionally, the results of the CAM assay confirmed that CLs could efficiently target and suppress the neovascularization indicating their potential to prevent tumor angiogenesis which merits further investigation. Although our MMP-2 responsive CLs showed acceptable efficacy against colon cancer, further work is still required to maximize their blood stability, selective biodistribution and tumor localization, dose adjustment, and cationic lipid/PEG ratio optimization. Additionally, replacement of the permanently charged cationic DOTAP in the formulation of these liposomes with the pH-sensitive ionizable amino-lipids that selectively become protonated at the lower pH of the tumor microenvironment or endosomes may restrict their lung accumulation. As such, the dilinoleylmethyl-4-dimethylaminobutyrate (DLin-MC3-DMA) in the formulation of the FDA-approved siRNA containing lipid nanoparticle product Onpattro™, or the lipid H (SM-102) or ALC-0315 in the Moderna and BioNTech/Pfizer COVID-19 vaccines, respectively, were used to increase their pH-dependent endosomal escape (Carrasco et al. 2021). These lipids have neutral charge at the physiologic blood pH but become protonated at the lower pH of endosomes that facilitates cytosolic delivery of their payloads. Intratumoral injection also remains as an alternative strategy to locally deliver CLs into the tumor tissue. Correspondingly, the CLs interaction with anionic structures of the tumor microenvironment after localized delivery may result in long tumor retention times bypassing non-specific systemic distribution and side effects (Han et al. 2014). Furthermore, dosing schedules should be optimized for desirable antitumor efficacy. It was previously reported that sequential treatment strategies of oxaliplatin-containing PEG-coated CLs led to superior tumor distribution and apoptosis in contrast to its mono-treatment regimen where their intratumoral localization was restricted to the areas of blood vessels (Lila et al. 2012). Thereby, the fraction of the chemotherapeutic drug delivered to the tumor tissue via CLs after a single injection might be insufficient to induce their potent therapeutic outcome which requires further investigations.

## Materials and methods

The full list of chemicals, reagents and cell lines is provided in the Additional file 1.

### Two-step synthesis of the mPEG2000-MMP2-cleavable peptide-DOPE conjugate (mPEG-peptide-DOPE)

The mPEG-peptide-DOPE conjugate was prepared using a procedure as elucidated in the Scheme 2. First, the MMP2-cleavable peptide (NH_2_-Gly-Pro-Leu-Gly-Ile-Ala-Gly-Gln-COOH) was mixed with mPEG-NHS (1:5 molar ratio) in phosphate buffer (pH 7.4) and stirred overnight at 2–8 °C. Thin-layer chromatography (TLC) was used for assessment of the reaction completion, and visualized using Dragendorff's or ninhydrin reagents for staining of the PEG chains or peptides, respectively. The reaction mixture was purified by dialysis through a 2 kDa molecular weight cutoff (MWCO) regenerated cellulose membrane (Spectrum Chemical Mfg. Corp, USA) against distilled water. The mPEG2000-peptide synthesis efficiency was evaluated using high performance liquid chromatography (HPLC) through a reverse phase C18 column (250 mm × 4.6 mm, Alltech, USA) on a prominence UFLC HPLC system (Shimadzu, Japan). The chromatograms were collected at 220 nm. HPLC grade acetonitrile/water (25:75, v/v) was used as the mobile phase in an isocratic elution mode at 25 °C with a 1 mL/min flow rate. The final product was lyophilized and stored at − 80 °C for further use.

In order to activate the mPEG2000-peptide (0.135 mmol), 1,3-dicyclohexyl-carbodiimide (DCC, 0.27 mmol), and 1-hydroxybenzotriazole (HOBt, 0.27 mmol) were added to its solution in chloroform and stirred under dry nitrogen for 2 h in an ice water bath. Next, DOPE (0.162 mmol) was dissolved in chloroform, mixed with the reaction mixture, and kept under dry nitrogen at 25 °C for 48 h. The completion of reaction was monitored using TLC, followed by the iodine staining. The final product was characterized by ^1^H nuclear magnetic resonance (NMR) spectroscopy in deuterated chloroform (CDCl_3_) at 400 MHz using Varian Mercury 400 (Varian Medical Systems Inc., Palo Alto, CA, USA).

### Preparation and Dox loading of the MMP-2 cleavable liposomes

Several liposomal formulations were fabricated using ethanol injection method as previously performed (Nikoofal-Sahlabadi et al. 2018). The lipid phase consisted of variable molar ratios of the cationic lipid DOTAP, and/or other neutral lipids including DOPE, HSPC, DSPE-mPEG2000, the MMP-2 cleavable mPEG2000-peptide-DOPE, cholesterol and the antioxidant α-tocopherol (Table 1). The total lipid concentration of each formulation was set to 50 mM. Briefly, lipids were dissolved in chloroform and mixed at different molar ratios in a round-bottom flask, as shown in Table 1. The organic solvent was then evaporated by a rotary evaporator (Heidolph, Germany), followed by a 2 h freeze-drying process using the VD-800F lyophilizer (Taitech, Koshigaya, Japan) to remove any residue of the organic solvent in the prepared thin layer lipid film. Next, the lipid film was dissolved in warm absolute ethyl alcohol (65 °C) and injected with pre-heated ammonium sulfate solution (250 mM, 65 °C) at 1:9 v/v ratio. The liposomal formulations were incubated at 65 °C for 1 h while shaking on a vortex shaker. This was followed by a 5 min sonication using a bath sonicator (Bandelin electronic GmbH & Co. KG, Berlin, Germany). The resultant large multilamellar vesicles (MLVs) were then extruded (11×) using the thermobarrel extruder (Avestin, Inc., Ottava, Canada) through 200 nm, 100 nm, and 50 nm polycarbonate filters (Whatman, Maidstone, UK). The final products were dialyzed (cellulose membrane, 12–14 kDa MWCO, MilliporeSigma, Burlington, MA, USA) against dextrose histidine buffer (10 mM, pH 6.5) to remove free ammonium sulfate. To Dox encapsulation, the amount of 1 mg Dox per 12 µmol total lipid from Dox solution was loaded into liposomes after incubation at 65 °C for 1 h, and cooled to room temperature. Free Dox were finally removed by dialysis (cellulose membrane, 12–14 kDa MWCO, MilliporeSigma, Burlington, MA, USA) against dextrose histidine buffer (10 mM, pH 6.5).

The ultimate prepared liposomal formulations were sterilized using 0.22 µm syringe filters and stored at 2–8 °C. Liposomes characterization is explained in detail in the SI.

### In vitro release study

The release patterns of the liposomes were assessed in various buffer systems providing different pHs (5.5, 6.5, and 7.4) to mimic the pH of late endosomes, early endosomes/tumor microenvironment, and plasma, respectively at 37 °C. These included the 5% dextrose/10 mM phosphate buffer (pH 7.4), 5% dextrose/10 mM histidine buffer (pH 6.5), 5% dextrose/10 mM succinate buffer (pH 5.5), or 5% dextrose: FBS (1:1 v/v, pH 7.4). Briefly, 1 mL of each formulation was placed into a dialysis bag (cellulose membrane, 12–14 kDa MWCO, MilliporeSigma, Burlington, MA, USA) and soaked in the 100 mL release medium. At different time-intervals (0, 0.25, 0.5, 0.75, 1, 2, 4, 6, 12, and 24 h), 1 mL of the incubated medium was sampled and replaced with 1 mL of the fresh medium. Dox concentration of collected samples were analyzed using a spectrofluorometer (ex: 490/em: 585) as explained in the Additional file 1 and % cumulative release calculated from the amounts of Dox detected at time t to the total content of Dox in liposomes initially used × 100.

### MTT cell viability assay

The cytotoxicity effect of formulations and free Dox was assessed against C26 colon carcinoma, 4T1 mammary carcinoma, B16F10 melanoma, and the normal NIH-3T3 fibroblast cell lines. The defined number of cells (C26: 3500, 4T1: 4000, B16F0: 3500, and NIH-3T3: 5000 cells/well) were seeded in 96-well plates and incubated overnight at 37 °C. Cells were then treated with equivalent concentration of the free Dox or Dox liposomal formulations that were serially diluted with FBS free medium at seven doses of Dox (0–5 μg/mL), Caelyx^®^ (0–50 μg/mL), PEGylated liposomes (0–5 μg/mL) and non-PEGylated CLs (0–2 μg/mL) in triplicate, and incubated for 48 h at 37 °C. Blank liposomes were used as controls for corresponding concentrations. After 48 h incubation, the medium was replaced with 100 µl of the fresh FBS free medium containing MTT solution (10:1 v/v) at 37 °C and incubated for 4 h. The media was then replaced by 200 µL of DMSO, and the absorbance was assessed at 570 nm using a Multiskan Plus microplate reader (BioTek EL 800; BioTek Instruments GmbH, Bad Friedrichshall, Germany) (Nikoofal-Sahlabadi et al. 2018). The relative cell growth inhibition (R) was computed as follows:

$$R=1-\left(\frac{{A}_{\mathrm{test}}-{A}_{\mathrm{blank}}}{{A}_{\mathrm{control}}-{A}_{\mathrm{blank}}}\right).$$

*A*_test_ and *A*_control_ are defined as absorbances of the cells incubated with the sample test and negative control (culture medium), respectively. *A*_blank_ indicates the absorbance of the MTT solution in the cell-free wells. The sensitivity to the administered formulations was assessed by the concentration required to inhibit 50% cell growth (IC_50_) was calculated using the CalcuSyn Software V. 2.0 (Biosoft, Cambridge, UK). The test was done in triplicate.

### Cell binding and uptake analysis

The liposome-cell interaction was evaluated using flow cytometry. Briefly, 10^6^ C26 cells were seeded in 6-well plates in 1 mL culture medium and incubated at 37 °C. After overnight incubation, the cells were treated with an equivalent concentration of the liposomal formulations and free Dox (10 μg/mL) and placed at 37 °C or 4 °C for 3 h. Subsequently, cells were washed three times with ice-cold phosphate-buffered saline (PBS), detached with 0.1 mL of the Trypsin–EDTA solution, and centrifuged at 1500 rpm for 5 min. The cell pellets were washed 3 × with PBS and resuspended in 300 μL PBS containing 1% FBS. Finally, the mean fluorescence intensity (MF) was measured using the flow cytometer (BD FACSCalibur™, BD Biosciences, USA) as described before (Arabi et al. 2015).

### Animal studies

BALB/c mice (aged 4–6 weeks, 18–20 g) were obtained from the Pasteur Institute of Iran (Tehran, Iran). The mice were maintained in the animal house of the Pharmaceutical Research Center of Mashhad University of Medical Sciences with 12/12 h light/dark cycles at 21 ± 2 °C with free access to a standard food diet and water ad libitum. All animal work was approbated by the Institutional Ethical Committee and Research Advisory Committee of Mashhad University of Medical Sciences (Ethical number: IR.MUMS.SP.1396.199). All animal experiments and methods were complied with the relevant guidelines and regulations approved by the ethical committee and the Animal Research: Reporting of in Vivo Experiments (ARRIVE) and performed in accordance with the UK Animals (Scientific Procedures) Act, 1986.

### Pharmacokinetic study

Healthy male BALB/c mice (4–6-week-old) were used to evaluate the pharmacokinetic profile of the formulations after intravenous (i.v.) administration. The mice (4 per group) were injected with a single dose of free Dox (10 mg/kg) or liposomal formulations via the tail vein. Control mice received 200 µL of PBS. Blood was collected from each mouse via the retro-orbital plexus at 1, 3, 6, and 24 h post-injection in the heparinized tubes. The blood samples were centrifuged for 10 min at 14,000 rpm. The plasma was collected, and Dox was diluted using acidified isopropanol and kept overnight at − 20 °C. The amount of Dox in blood samples was then assayed using the Perkin-Elmer LS-45 spectrofluorometer (Ex/Em = 490/585 nm; Perkin-Elmer, Waltham, USA).

The pharmacokinetic study of different formulations was investigated using the non-compartmental analysis model. The maximum plasma concentration (*C*_max_), the area under the plasma concentration–time curve (AUC), the volume of distribution (*V*_d_), half-life (*t*_1/2_), clearance (Cl) and mean residence time (MRT) were determined as described before (Mashreghi et al. 2021). The test was performed in triplicate.

### Tumor induction

Tumor development was induced by subcutaneous injection of 60 μL PBS containing 3 × 10^5^ C26 cells in the right flank of female BALB/c mice. The criteria set out by the Institutional Ethical Committee and Research Advisory were followed in all the procedures.

### Biodistribution study

14 days after tumor inoculation, when the tumor size was approximately 5 mm wide, mice were randomly classified into 9 treatment groups (*n* = 3). The free Dox, Caelyx^®^, F2, F3, F4, F5, F6, F7, and F8 formulations were then systemically injected via the tail vein at the equivalent dose of 10 mg/kg of Dox. 200 µL of PBS were injected to control mice. 24 h later, animals were killed and their main organs, including spleen, lung, heart, kidney, a piece of liver, and the tumor region were removed, weighed, and put in a 2 mL polypropylene microvials (BioSpec Products, Inc., Bartlesville, USA) containing 1 mL of acidified isopropanol plus zirconia beads (1600 mg) and homogenized by the Mini-Beadbeater-1 (BioSpec Products, Inc., Bartlesville, USA). The homogenized tissue samples were then stored at 2–8 °C. The samples were finally centrifuged at 14,000 rpm for 10 min, and the Dox concentration in the supernatant was assessed using spectrofluorometry as described in the Additional file 1.

### Antitumor efficacy and survival analysis

Seven days post-inoculation of C26 tumor cells, when the tumors were palpable, the mice (*n* = 8 per group) were administrated with a single dose of 10 mg/kg of free Dox or the equivalent dose of liposomal formulations via the tail vein. The negative control group received PBS. Parameters of body weight and tumor volume (*V*_T_) were subsequently monitored every two days during the study. In order to calculate the *V*_T_, three diameters of tumors were measured using the Mitutoyo 500-196-20 digital caliper (Kanagawa, Japan), and *V*_T_ was calculated using the following formula: *V*_T_ = height × length × width × 0.5 cm^3^. The survival parameters, including median survival time (MST), percentage of tumor growth delay (%TGD), and time to reach the end (TTE), were measured as explained before (Huang et al. 2009).

Mice were euthanized in accordance with the ethical considerations when tumor development reached a *V*_T_ > 1000 mm^3^, or > 20% weight loss or symptoms of weakness were observed. On day 20, three mice from each group were killed and major organs including heart, lung, spleen, liver and kidney taken out, washed with 0.9% w/v NaCl solution and fixed in 10% (v/v) formalin. All samples were then embedded in paraffin blocks, sectioned into 5 µm in thickness and mounted on glass slides. Next, slides were stained with hematoxylin and eosin (H&E), and imaged utilizing an optical microscope.

### Statistical analysis

Data were statistically analyzed using GraphPad Prism 8.0 (GraphPad Software, Inc., San Diego, USA). Kaplan–Meier survival analysis was employed to compare antitumor activities of diverse groups. The significant differences between groups were compared using the two-way ANOVA analysis and the Tukey–Kramer post hoc analysis. A *P*-value < 0.05 was regarded statically significance (*).

## Supplementary Information

**Additional file 1: Table S1.** Stability of CL formulations during 8 months at 2-8 °C. **Table S2.** Pharmacokinetic parameters of the Dox-loaded liposomal formulations. **Table S3.** Antitumor effects of the Dox-loaded liposomal formulations. **Figure S1.** (A) HPLC analysis data for the free MMP-2 cleavable peptide with a retention time of 3.25 min and the mPEG-peptide-DOPE conjugate with a retention time of 2.6 min revealed the consumption of free peptide in the reaction and formation of the conjugate. B) Dragendorff's reagent and C) Ninhydrin reagent staining of the PEG chains and the peptide. **Figure S2.** Final product was confirmed by iodine staining of the TLC plate. (1) DOPE, (2) PEG, (3) m-PEG-peptide-DOPE. **Figure S3.** ^1^HNMR spectra of the DOPE (black), MMP-2 cleavable peptide (blue) or the PEG-peptide-DOPE conjugate (reddish-brown). **Figure S4.** Biodistribution of the free Dox and Dox-loaded liposomes after 24 h (A) and their therapeutic efficacy in male BALB/c mice bearing C26 tumor [survival curve (B), tumor volume (C) and body weight (D)]. **Figure S5.** Tumor volume of the C26-bearing mice treated with different liposomal formulations.

## Data Availability

Data will be made available on request.

## References

[CR1] Abu Lila AS, Ishida T, Kiwada H (2010). Targeting anticancer drugs to tumor vasculature using cationic liposomes. Pharm Res.

[CR2] Abu-Lila A, Suzuki T, Doi Y, Ishida T, Kiwada H (2009). Oxaliplatin targeting to angiogenic vessels by PEGylated cationic liposomes suppresses the angiogenesis in a dorsal air sac mouse model. J Control Release.

[CR3] Arabi L, Badiee A, Mosaffa F, Jaafari MR (2015). Targeting CD44 expressing cancer cells with anti-CD44 monoclonal antibody improves cellular uptake and antitumor efficacy of liposomal doxorubicin. J Control Release.

[CR4] Awada A, Bondarenko I, Bonneterre J, Nowara E, Ferrero J, Bakshi A (2014). A randomized controlled phase II trial of a novel composition of paclitaxel embedded into neutral and cationic lipids targeting tumor endothelial cells in advanced triple-negative breast cancer (TNBC). Ann Oncol.

[CR5] Bahari LAS, Hamishehkar H (2016). The impact of variables on particle size of solid lipid nanoparticles and nanostructured lipid carriers; a comparative literature review. Adv Pharmaceut Bull.

[CR6] Barenholz YC (2012). Doxil^®^—the first FDA-approved nano-drug: lessons learned. J Control Release.

[CR7] Campbell RB, Ying B, Kuesters GM, Hemphill R (2009). Fighting cancer: from the bench to bedside using second generation cationic liposomal therapeutics. J Pharm Sci.

[CR8] Carrasco MJ, Alishetty S, Alameh M-G, Said H, Wright L, Paige M (2021). Ionization and structural properties of mRNA lipid nanoparticles influence expression in intramuscular and intravascular administration. Commun Biol.

[CR9] Dass CR (2003). Improving anti-angiogenic therapy via selective delivery of cationic liposomes to tumour vasculature. Int J Pharm.

[CR10] Dicheva BM, Hagen TL, Li L, Schipper D, Seynhaeve AL, Rhoon GC (2013). Cationic thermosensitive liposomes: a novel dual targeted heat-triggered drug delivery approach for endothelial and tumor cells. Nano Lett.

[CR11] Dicheva BM, ten Hagen TL, Schipper D, Seynhaeve AL, van Rhoon GC, Eggermont AM (2014). Targeted and heat-triggered doxorubicin delivery to tumors by dual targeted cationic thermosensitive liposomes. J Control Release.

[CR12] Fang Y, Xue J, Gao S, Lu A, Yang D, Jiang H (2017). Cleavable PEGylation: a strategy for overcoming the “PEG dilemma” in efficient drug delivery. Drug Deliv.

[CR13] Gabizon A, Shmeeda H, Barenholz Y (2003). Pharmacokinetics of pegylated liposomal doxorubicin. Clin Pharmacokinet.

[CR14] Haghiralsadat F, Amoabediny G, Helder MN, Naderinezhad S, Sheikhha MH, Forouzanfar T (2018). A comprehensive mathematical model of drug release kinetics from nano-liposomes, derived from optimization studies of cationic PEGylated liposomal doxorubicin formulations for drug-gene delivery. Artif Cells Nanomed Biotechnol.

[CR15] Han HD, Byeon Y, Jeon HN, Shin BC (2014). Enhanced localization of anticancer drug in tumor tissue using polyethylenimine-conjugated cationic liposomes. Nanosc Res Lett.

[CR16] Hattori Y, Saito H, Oku T, Ozaki K-I (2021). Effects of sterol derivatives in cationic liposomes on biodistribution and gene-knockdown in the lungs of mice systemically injected with siRNA lipoplexes. Mol Med Rep.

[CR17] Huang Z, Jaafari MR, Szoka FC (2009). Disterolphospholipids: nonexchangeable lipids and their application to liposomal drug delivery. Angew Chem.

[CR18] Hwang T, Han HD, Song CK, Seong H, Kim JH, Chen X (2007). 'Anticancer drug-phospholipid conjugate for enhancement of intracellular drug delivery. Macromolecular symposia.

[CR19] Jung SH, Jung SH, Seong H, Cho SH, Jeong K-S, Shin BC (2009). Polyethylene glycol-complexed cationic liposome for enhanced cellular uptake and anticancer activity. Int J Pharm.

[CR20] Karimi M, Gheybi F, Zamani P, Mashreghi M, Golmohammadzadeh S, Darban SA (2020). Preparation and characterization of stable nanoliposomal formulations of curcumin with high loading efficacy: in vitro and in vivo anti-tumor study. Int J Pharm.

[CR21] Kuipers E, Grady W, Lieberman D, Seufferlein T, Sung J, Boelens P (2015). Colorectal cancer. Nat Rev Dis Primers.

[CR22] Li J, Wang X, Zhang T, Wang C, Huang Z, Luo X (2015). A review on phospholipids and their main applications in drug delivery systems. Asian J Pharm Sci.

[CR23] Lila ASA, Kizuki S, Doi Y, Suzuki T, Ishida T, Kiwada H (2009). Oxaliplatin encapsulated in PEG-coated cationic liposomes induces significant tumor growth suppression via a dual-targeting approach in a murine solid tumor model. J Control Release.

[CR24] Lila ASA, Eldin NE, Ichihara M, Ishida T, Kiwada H (2012). Multiple administration of PEG-coated liposomal oxaliplatin enhances its therapeutic efficacy: a possible mechanism and the potential for clinical application. Int J Pharm.

[CR25] Liu C, Zhang L, Zhu W, Guo R, Sun H, Chen X (2020). Barriers and strategies of cationic liposomes for cancer gene therapy. Mol Thera Methods Clin Dev.

[CR26] Löhr J, Haas S, Bechstein W-O, Bodoky G, Cwiertka K, Fischbach W (2012). Cationic liposomal paclitaxel plus gemcitabine or gemcitabine alone in patients with advanced pancreatic cancer: a randomized controlled phase II trial. Ann Oncol.

[CR27] Ma M, Lei M, Tan X, Tan F, Li N (2016). Theranostic liposomes containing conjugated polymer dots and doxorubicin for bio-imaging and targeted therapeutic delivery. RSC Adv.

[CR28] Maleki MF, Jafari A, Mirhadi E, Askarizadeh A, Golichenari B, Hadizadeh F (2019). Endogenous stimuli-responsive linkers in nanoliposomal systems for cancer drug targeting. Int J Pharm.

[CR29] Mashreghi M, FaalMaleki M, Karimi M, Kalalinia F, Badiee A, Jaafari MR (2021). Improving anti-tumour efficacy of PEGylated liposomal doxorubicin by dual targeting of tumour cells and tumour endothelial cells using anti-p32 CGKRK peptide. J Drug Target.

[CR30] Mirafzali Z, Thompson CS, Tallua K (2014). Application of liposomes in the food industry. Microencapsulation in the food industry.

[CR31] Mochizuki S, Kanegae N, Nishina K, Kamikawa Y, Koiwai K, Masunaga H (2013). The role of the helper lipid dioleoylphosphatidylethanolamine (DOPE) for DNA transfection cooperating with a cationic lipid bearing ethylenediamine. Biochim Biophys Acta BBA Biomembr.

[CR32] Nakhaei P, Margiana R, Bokov DO, Abdelbasset WK, Kouhbanani MAJ, Varma RS (2021). Liposomes: structure, biomedical applications, and stability parameters with emphasis on cholesterol. Front Bioeng Biotechnol.

[CR33] Nik ME, Malaekeh-Nikouei B, Amin M, Hatamipour M, Teymouri M, Sadeghnia HR (2019). Liposomal formulation of Galbanic acid improved therapeutic efficacy of pegylated liposomal Doxorubicin in mouse colon carcinoma. Sci Rep.

[CR34] Nikoofal-Sahlabadi S, Riahi MM, Sadri K, Badiee A, Nikpoor AR, Jaafari MR (2018). Liposomal CpG-ODN: an in vitro and in vivo study on macrophage subtypes responses, biodistribution and subsequent therapeutic efficacy in mice models of cancers. Eur J Pharm Sci.

[CR35] Niu G, Castro CH, Nguyen N, Sullivan SM, Hughes JA (2010). In vitro cytotoxic activity of cationic paclitaxel nanoparticles on MDR-3T3 cells. J Drug Target.

[CR36] Olsman M, Sereti V, Andreassen K, Snipstad S, van Wamel A, Eliasen R (2020). Ultrasound-mediated delivery enhances therapeutic efficacy of MMP sensitive liposomes. J Control Release.

[CR37] Park J-H, Cho H-J, Yoon HY, Yoon I-S, Ko S-H, Shim J-S (2014). Hyaluronic acid derivative-coated nanohybrid liposomes for cancer imaging and drug delivery. J Control Release.

[CR38] Parr MJ, Masin D, Cullis PR, Bally MB (1997). Accumulation of liposomal lipid and encapsulated doxorubicin in murine Lewis lung carcinoma: the lack of beneficial effects by coating liposomes with poly (ethylene glycol). J Pharmacol Exp Ther.

[CR39] Piperigkou Z, Kyriakopoulou K, Koutsakis C, Mastronikolis S, Karamanos NK (2021). Key matrix remodeling enzymes: functions and targeting in cancer. Cancers.

[CR40] Pozzi D, Colapicchioni V, Caracciolo G, Piovesana S, Capriotti AL, Palchetti S (2014). Effect of polyethyleneglycol (PEG) chain length on the bio-nano-interactions between PEGylated lipid nanoparticles and biological fluids: from nanostructure to uptake in cancer cells. Nanoscale.

[CR41] Ray S, Cheng C, Chen W, Li Z, Zink J, Lin Y (2019). Magnetic heating stimulated cargo release with dose control using multifunctional MR and thermosensitive liposome. Nanotheranostics..

[CR42] Ribatti D, Annese T, Tamma R (2020). The use of the chick embryo CAM assay in the study of angiogenic activiy of biomaterials. Microvasc Res.

[CR43] Samuelsson E, Shen H, Blanco E, Ferrari M, Wolfram J (2017). Contribution of Kupffer cells to liposome accumulation in the liver. Colloids Surf B.

[CR44] Sasayama Y, Hasegawa M, Taguchi E, Kubota K, Kuboyama T, Naoi T (2019). In vivo activation of PEGylated long circulating lipid nanoparticle to achieve efficient siRNA delivery and target gene knock down in solid tumors. J Control Release.

[CR45] Şen Ö, Emanet M, Ciofani G (2021). Nanotechnology-based strategies to evaluate and counteract cancer metastasis and neoangiogenesis. Adv Healthc Mater.

[CR46] Sharifi S, Caracciolo G, Mahmoudi M (2020). Biomolecular corona affects controlled release of drug payloads from nanocarriers. Trends Pharmacol Sci.

[CR47] Song H, Wei M, Zhang N, Li H, Tan X, Zhang Y (2018). Enhanced permeability of blood–brain barrier and targeting function of brain via borneol-modified chemically solid lipid nanoparticle. Int J Nanomed.

[CR48] Sonowal H, Pal PB, Wen J-J, Awasthi S, Ramana KV, Srivastava SK (2017). Aldose reductase inhibitor increases doxorubicin-sensitivity of colon cancer cells and decreases cardiotoxicity. Sci Rep.

[CR49] Sopyan I, Gozali D (2020) A review: a novel of efforts to enhance liposome stability as drug delivery approach. Syst Rev Pharm 11(6)

[CR50] Sultana S, Alzahrani N, Alzahrani R, Alshamrani W, Aloufi W, Ali A (2020). Stability issues and approaches to stabilised nanoparticles based drug delivery system. J Drug Target.

[CR51] Sung H, Ferlay J, Siegel RL, Laversanne M, Soerjomataram I, Jemal A (2021). Global cancer statistics 2020: GLOBOCAN estimates of incidence and mortality worldwide for 36 cancers in 185 countries. CA Cancer J Clin.

[CR52] Terada T, Iwai M, Kawakami S, Yamashita F, Hashida M (2006). Novel PEG-matrix metalloproteinase-2 cleavable peptide-lipid containing galactosylated liposomes for hepatocellular carcinoma-selective targeting. J Control Release.

[CR53] Thurston G, McLean JW, Rizen M, Baluk P, Haskell A, Murphy TJ (1998). Cationic liposomes target angiogenic endothelial cells in tumors and chronic inflammation in mice. J Clin Investig.

[CR54] van der Zanden SY, Qiao X, Neefjes J (2021). New insights into the activities and toxicities of the old anticancer drug doxorubicin. FEBS J.

[CR55] Wang L, Zhang T-P, Zhang Y, Bi H-L, Guan X-M, Wang H-X (2016). Protection against doxorubicin-induced myocardial dysfunction in mice by cardiac-specific expression of carboxyl terminus of hsp70-interacting protein. Sci Rep.

[CR56] Wang W, Shao A, Zhang N, Fang J, Ruan JJ, Ruan BH (2017). Cationic polymethacrylate-modified liposomes significantly enhanced doxorubicin delivery and antitumor activity. Sci Rep.

[CR57] Wibroe PP, Ahmadvand D, Oghabian MA, Yaghmur A, Moghimi SM (2016). An integrated assessment of morphology, size, and complement activation of the PEGylated liposomal doxorubicin products Doxil^®^, Caelyx^®^, DOXOrubicin, and SinaDoxosome. J Control Release.

[CR58] Yao M, Ma X, Zhang X, Shi L, Liu T, Liang X (2019). Lectin-mediated pH-sensitive doxorubicin prodrug for pre-targeted chemotherapy of colorectal cancer with enhanced efficacy and reduced side effects. Theranostics.

[CR59] Zhao W, Zhuang S, Qi X-R (2011). Comparative study of the in vitro and in vivo characteristics of cationic and neutral liposomes. Int J Nanomed.

[CR60] Zhao C, Deng H, Xu J, Li S, Zhong L, Shao L (2016). “Sheddable” PEG-lipid to balance the contradiction of PEGylation between long circulation and poor uptake. Nanoscale.

[CR61] Zhao Y, Ren W, Zhong T, Zhang S, Huang D, Guo Y (2016). Tumor-specific pH-responsive peptide-modified pH-sensitive liposomes containing doxorubicin for enhancing glioma targeting and anti-tumor activity. J Control Release.

[CR62] Zhu L, Kate P, Torchilin VP (2012). Matrix metalloprotease 2-responsive multifunctional liposomal nanocarrier for enhanced tumor targeting. ACS Nano.

[CR63] Zhu Y, Meng Y, Zhao Y, Zhu J, Xu H, Zhang E (2019). Toxicological exploration of peptide-based cationic liposomes in siRNA delivery. Colloids Surf B.

