# Supplementary material for: Doxorubicin-loaded liposomes surface engineered with the matrix metalloproteinase-2 cleavable polyethylene glycol conjugate for cancer therapy
Source: Cancer Nanotechnol. 2023 Mar 7;14(1):18. doi: 10.1186/s12645-023-00169-8 (PMC9988605; doi:10.1186/s12645-023-00169-8)
Supplement: Supplementary file 1 — Additional file 1: Table S1. Stability of CL formulations during 8 months at 2-8 °C. Table S2. Pharmacokinetic parameters of the Dox-loaded liposomal formulations. Table S3. Antitumor effects of the Dox-loaded liposomal formulations. Figure S1. (A) HPLC analysis data for the free MMP-2 cleavable peptide with a retention time of 3.25 min and the mPEG-peptide-DOPE conjugate with a retention time of 2.6 min revealed the consumption of free peptide in the reaction and formation of the conjugate. B) Dragendorff's reagent and C) Ninhydrin reagent staining of the PEG chains and the peptide. Figure S2. Final product was confirmed by iodine staining of the TLC plate. (1) DOPE, (2) PEG, (3) m-PEG-peptide-DOPE. Figure S3. 1HNMR spectra of the DOPE (black), MMP-2 cleavable peptide (blue) or the PEG-peptide-DOPE conjugate (reddish-brown). Figure S4. Biodistribution of the free Dox and Dox-loaded liposomes after 24 h (A) and their therapeutic efficacy in male BALB/c mice bearing C26 tumor [survival curve (B), tumor volume (C) and body weight (D)]. Figure S5. Tumor volume of the C26-bearing mice treated with different liposomal formulations. [file 12645_2023_169_MOESM1_ESM.docx]

**SUPPLEMENTARY INFORMATION**

**Doxorubicin-loaded liposomes surface engineered with the matrix metalloproteinase-2 cleavable polyethylene glycol conjugate for cancer therapy**

Anis Askarizadeh^1,2^, Mohammad Mashreghi^1,2^, Elaheh Mirhadi^1,2^, Farshad Mirzavi^3^, Vahid Heravi Shargh^4 *^, Ali Badiee^1,2^, Seyedeh Hoda Alavizadeh^1,2^, Leila Arabi^1,2^, Mahmoud Reza Jaafari^1,2,*^

^1^ Nanotechnology Research Center, Pharmaceutical Technology Institute, Mashhad University of Medical Sciences, Mashhad, Iran; ^2^ Department of Pharmaceutical Nanotechnology, School of Pharmacy, Mashhad University of Medical Sciences, Mashhad, Iran; ^3^ Cardiovascular Diseases Research Center, Birjand University of Medical Sciences, Birjand, Iran; ^4^ Division of Pharmacy and Optometry, Faculty of Biology, Medicine and Health, University of Manchester, Manchester, UK

^*^ Correspondence: jafarimr@mums.ac.ir; Mashhad University of Medical Sciences, Mashhad, Iran; Tel.: +98 513 1801336; Fax: +98 513 8823251.

vahid.heravishargh@manchester.ac.uk; Division of Pharmacy and Optometry, School of Health Sciences, Faculty of Biology, Medicine and Health, The University of Manchester, Manchester, UK, Tel: +447741185285

**Materials and Methods**

All lipid including the 1,2-dioleoyl-sn-glycero-3-phosphoethanolamine (DOPE), 1,2-dioleoyl-3-trimethylammonium-propane (DOTAP), 1,2-distearoyl-sn-glycero-3-phosphoethanolamine-N-[methoxy(polyethylene glycol)-2000] (DSPE-mPEG2000), and hydrogenated soy phosphatidylcholine (HSPC) were purchased from the Lipoid GmbH (Ludwigshafen, Germany). Cholesterol, Dox hydrochloride, and ammonium sulfate were obtained from MilliporeSigma (Burlington, MA, USA). N-hydroxylsuccinimide (NHS)-functionalized polyethylene glycol (mPEG-NHS; average MW: 2000 Da) was procured from the Laysan Bio, Inc. (Arab, AL, USA). MMP-2 peptide at 99.9% purity was obtained from the Chinese Peptide Company (Hangzhou Economic and Technological Development Zone, China). Trypan blue, streptomycin, penicillin, RPMI 1640 (Roswell Park Memorial Institute medium), DMEM (Dulbecco's Modified Eagle's medium) culture media and fetal bovine serum (FBS) were purchased from Thermo Fisher Scientific (Waltham, MA, USA). 3-(4,5-dimethylthiazol-2-yl)-2,5-diphenyltetrazolium bromide (MTT) was obtained from Promega (Madison, WI, USA). The commercially available PEGylated liposomal Dox (Caelyx^®^) was supplied from Behestan Darou Company. All other solvents and reagents were of chemical grade. C26 murine colon carcinoma, 4T1 murine breast adenocarcinoma, B16F10 murine melanoma and NIH-3T3 embryonic fibroblast cell lines were obtained from the Pasteur Institute of Iran (Tehran, Iran).

**Liposomes characterization**

The hydrodynamic diameter, polydispersity index (PDI) and zeta potential of nanoparticles were determined using Dynamic Light Scattering (DLS, NANO-ZS, Malvern, UK) (Riahi et al., 2018). The phospholipid content of liposomes was assessed through the Bartlett phosphate test (Riahi et al., 2018). To evaluate the morphological characteristic of liposomes, negative staining transmission electron microscopy (TEM) was implemented. The sample preparation for TEM photography was performed in this way; first, liposomes were diluted (1:10 dilution of liposomes in dialysis buffer), and 20 µl of the sample was dropped onto a carbon-coated copper grid. After 1 minute, the surplus liposomes were cleared by a filter paper. In the next step, 20 µl filtered uranyl acetate (2% w/v) was placed onto the grid. After staining, the samples were observed and a ZEISS LEO 912 TEM at an accelerating voltage of 80 kV (Jena, Germany) was used to sample photography (Karimi et al., 2020).

To evaluate the amount of Dox in the purified liposomes, aliquots of Dox-loaded liposomal formulations were dissolved in acidified isopropyl alcohol (90% isopropanol, 0.075 M HCl). Dox concentration was then determined by a spectrofluorometer (ex: 490/em: 585, Shimadzu RF5000U, Kyoto, Japan). The percentage of encapsulation efficiency (EE%) of Dox was then calculated by the difference between the total amount of Dox initially added during the preparation process and the amount of entrapped Dox in the final product, as shown in the following equation:

EE% = (mg DOX in dialyzed liposomes/mg DOX initially added) × 100

**Analysis of the physicochemical stability of liposomes**

The physical stability of Dox liposomal formulations was monitored while incubated in sealed vials and protected from light at 4 °C. For this purpose, at different time intervals (0, 2, 8, and 14-months post-preparation), samples were collected and evaluated in terms of appearance, particle size, EE%, Dox leakage, and the tendency to form precipitates. As a result, the leakage ratio of Dox was determined by comparing its EE% at each time point with the EE% of the same batch at the starting point of the test.

**RNA extraction and quantitative real-time PCR (qRT-PCR)**

Total RNA was extracted from the NIH-3T3, C26, 4T1, and B16F10 cells using the Total RNA Extraction Kit (Pars Tous Co., Iran). The purity and concentration of isolated RNAs were evaluated by NanoDrop ND-1000 (Thermo Fischer Scientific). Subsequently, RNAs were reverse-transcribed to complementary DNA (cDNA) by a commercially available cDNA synthesis kit (Pars Tous Co., Iran). Next, a LightCycler® 96 RT-PCR system (Roche, Basel, Switzerland‎) was used for the qRT-PCR amplifications of MMP-2.

MMP-2, 5′-GGTGCTCCACTCTTCTGGTT-3′ (forward) and 5′-ATTTCCACCTTGGCACACCT-3′ (reverse); glyceraldehyde-3-phosphate dehydrogenase (GAPDH), 5′-CAACGACCCCTTCATTGACC-3′ (forward) and 5′-CTTCCCATTCTCGGCCTTGA-3′ (reverse) were selected as specific primers. GAPDH was applied as an internal reference gene and fold change gene expression was estimated with the 2^-ΔΔCT^ method (Livak and Schmittgen, 2001).

**Gelatin zymography**

The MMP-2 activity was measured using gelatin zymography in the NIH-3T3, C26, 4T1, and B16F10 cell lines (Mirzavi et al., 2022). Briefly, media supernatants of cultured cells (50 µg protein) were loaded onto 10% separating sodium dodecyl sulfate-polyacrylamide gel electrophoresis (SDS-PAGE) containing 0.1% w/v of gelatin. Next, the gel washing was conducted three times using 2.5% v/v Triton X-100 containing distilled water for 20-min and then incubated in the 50 mM developing buffer containing 2.5% Triton X-100, 5 mM CaCl2, and 1 μM ZnCl2 (pH 7.4) for 48 h at 37 °C. Next, the gel staining was carried out using 0.5% w/v Coomassie Brilliant Blue R-250 dispersed in distilled water containing 25% v/v methanol and 10% v/v acetic acid for 1 h at 25 °C and subsequently incubated with destaining solution (40% v/v methanol and 10% v/v acetic acid) until white bands could be detected. To scan the zones of gelatinase activity and evaluate images, GS-800TM calibrated densitometer (Bio-RAD, USA) and ImageJ 1.52a software were applied respectively.

**Evaluation of the intracellular uptake of Dox using fluorescence microscopy**

In each well of a 6-well plate, sterile coverslips were inserted, and 3 × 10^5^ C26 cells were seeded per well. After 24 h of incubation, the cells were treated with an equivalent concentration of the free Dox or Dox-loaded liposomes (10 μg/mL) and incubated at 37 °C for 3 h. Next, cells were washed with PBS (3x) and fixed with 4% paraformaldehyde for 10 min. Subsequently, cells were washed with PBS and incubated with the DAPI labeling solution in the dark. Coverslips were finally placed on a microscope slide and cellular uptake visualized by the intrinsic fluorescence of Dox using the Olympus BX-51 fluorescence microscope (Olympus, Japan) (Mirhadi et al., 2022).

**Antiangiogenic activity**

A chick chorioallantoic membrane (CAM) experiment was used to assess the antiangiogenic effectiveness of liposomal formulations (Nik et al., 2019). Fertilized chick eggs were first maintained in a Multiquip E2 incubator (Multiquip, Cypress, USA) at 37 °C with 60% humidity. On day eight and under aseptic conditions, the eggshell was partially opened (1 × 1 cm), and 20 µl of each formulation were injected into the CAM. The window was covered by the parafilm and fixed with adhesive tape. As a negative control, only PBS was injected into the CAM. On day 12, the window opened and vasculature of the CAM was photographed with a Luxeo 4Z stereo zoom microscope (Labomed, Los Angeles, USA), and the pictures were analyzed by an ImageJ angiogenesis analyzer plugin.

Table S1. Stability of CL formulations during 8 months at 2-8 °C

| **EE%^c^** | **Zeta-potential (mV) ± SD** | **PDI^b^ ± SD** | **Size (nm) ± SD^a^** | **Time (month)** | **Formulation** |
| --- | --- | --- | --- | --- | --- |
| 100 | -10.1 ± 0.2 | 0.10 ± 0.04 | 92.3 ± 4.2 | 0 | F1  HSPC/Chol/DSPE-PEG2000/α-tocopherol (56.7/38/5.3/0.2) |
| 100 | -9.5 ± 1.4 | 0.12 ± 0.03 | 92.9 ± 4.2 | 1 |  |
| 99 | -12.9 ± 0.7 | 0.14 ± 0.007 | 95.1 ± 2.23 | 4 |  |
| 98 | -14.1 ± 0.3 | 0.15 ± 0.02 | 97.1 ± 3.56 | 8 |  |
| 94 | -7.6 ± 0.2 | 0.19 ± 0.02 | 159.5 ± 3.5 | 0 | F2  HSPC/Chol/DOPE-peptide-mPEG/α-tocopherol (59.5/38/2.5/0.2) |
| 91 | -7.1 ± 0.1 | 0.19 ± 0.01 | 162.04 ± 2.1 | 1 |  |
| 83.5 | -8.4 ± 0.8 | 0.21 ± 0.3 | 171.5 ± 1.9 | 4 |  |
| 70 | -10.3 ± 2.6 | 0.24 ± 0.01 | 192.5 ± 2.6 | 8 |  |
| 90 | -5.3 ± 0.3 | 0.19 ± 0.03 | 160.7 ± 1.9 | 0 | F3  HSPC/Chol/DOPE-peptide-mPEG/α-tocopherol (57/38/5/0.2) |
| 84.2 | -5.8 ± 0.4 | 0.18 ± 0.01 | 164.9 ± 3.5 | 1 |  |
| 71.6 | -7.5 ± 0.6 | 0.25 ± 0.02 | 173.4 ± 4.6 | 4 |  |
| 60.4 | -11.4 ± 0.2 | 0.31 ± 0.04 | 189.6 ± 2.9 | 8 |  |
| 88 | +17.6 ± 0.2 | 0.13 ± 0.01 | 127.1 ± 1.3 | 0 | F4  DOTAP/HSPC/Chol/DOPE/α-tocopherol (10/50/38/2/0.2) |
| 87.8 | +17.1 ± 0.4 | 0.12 ± 0.02 | 127.6 ± 2.5 | 1 |  |
| 79.6 | +16.6 ± 0.2 | 0.12 ± 0.01 | 131.7 ± 3 | 4 |  |
| 74 | +16.1 ± 0.3 | 0.13 ± 0.03 | 135 ± 4.1 | 8 |  |
| 95 | +14.9 ± 0.1 | 0.12 ± 0.04 | 130.9 ± 2.2 | 0 | F5  DOTAP/HSPC/Chol/DOPE-peptide-PEG/α-tocopherol (10/49.5/38/2.5/0.2) |
| 93.8 | +14.8 ± 0.2 | 0.12 ± 0.01 | 128.6 ± 3.8 | 1 |  |
| 86.4 | +14.4 ± 0.3 | 0.13 ± 0.05 | 138.4 ± 4 | 4 |  |
| 84.4 | +13.9 ± 0.2 | 0.15 ± 0.04 | 147.6 ± 1.7 | 8 |  |
| 84 | +10.3 ± 0.4 | 0.16 ± 0.02 | 124.7 ± 4.1 | 0 | F6  DOTAP/HSPC/Chol/DOPE-peptide-PEG/α-tocopherol (10/47/38/5/0.2) |
| 80.43 | +10.3 ± 0.5 | 0.16 ± 0.01 | 125.4 ± 5.2 | 1 |  |
| 75 | +11.5 ± 0.2 | 0.17 ± 0.04 | 133.8 ± 3.2 | 4 |  |
| 72.3 | +12.8 ± 0.1 | 0.18 ± 0.01 | 140.3 ± 2.5 | 8 |  |
| 92 | +13.6 ± 0.1 | 0.11 ± 0.01 | 120.8 ± 3.1 | 0 | F7  DOTAP/HSPC/Chol/DOPE/DSPE-PEG2000/ α-tocopherol (10/47/38/2.5/2.5/0.2) |
| 91.5 | +13 ± 0.2 | 0.11 ± 0.03 | 120.5 ± 2.7 | 1 |  |
| 87.7 | +13.3 ± 0.3 | 0.13 ± 0.05 | 128 ± 4.4 | 4 |  |
| 84 | +12.9 ± 0.1 | 0.16 ± 0.02 | 142.6 ± 1.9 | 8 |  |
| 80 | +3.67 ± 0.3 | 0.18 ± 0.04 | 170.3 ± 2.9 | 0 | F8  DOTAP/HSPC/Chol/DOPE/DSPE-PEG2000/α-tocopherol (10/42/38/5/5/0.2) |
| 74.4 | +4.7 ± 0.8 | 0.18 ± 0.01 | 172 ± 3.5 | 1 |  |
| 68 | +4.1 ± 0.6 | 0.20 ± 0.03 | 188.4 ± 4.7 | 4 |  |
| 60.34 | +3.8 ± 0.4 | 0.23 ± 0.02 | 206.9 ± 2.4 | 8 |  |

^a^ Diameter of liposomes (Z average); ^b^ Polydispersity index; ^c^ Encapsulation efficacy. Data are presented from triplicate measurements of each formulation as mean ± standard deviation (SD).

Table S2. Pharmacokinetic parameters of the Dox-loaded liposomal formulations

| **MRT**  **(h)** | **AUMC**  **(µg*h^2^/mL)** | **AUC**  **(µg*h/mL)** | **Cl**  **(mg)/(µg/mL)/h** | **V_ss_**  **(mg)/**  **(µg/mL)** | **t_1/2_**  **(h)** | **K**  **(1/h)** | **Treatment group** |
| --- | --- | --- | --- | --- | --- | --- | --- |
| 23.08 | 24563.25 | 1064.08 | 0.0094 | 0.21 | 18.06 | 0.038 | **F2** |
| 8.82 | 3347.99 | 379.411 | 0.0263 | 0.23 | 7.73 | 0.089 | **F3** |
| 11.96 | 6724.17 | 562.07 | 0.017 | 0.21 | 9.24 | 0.074 | **F7** |
| 20.37 | 11090.68 | 544.353 | 0.018 | 0.37 | 14.13 | 0.049 | **F8** |

Table S3. Antitumor effects of the Dox-loaded liposomal formulations.

| **Formulation** | **Time to reach the end point (days)** | **Tumor growth**  **delay (TGD%)** | **Median**  **survival time**  **(days)** | **Percentage increased life span (ILS%)** |
| --- | --- | --- | --- | --- |
| F2 | 58.1 ± 4.1 | 70.46 | 57.4 | 71.94 |
| F3 | 48.8 ± 2.8 | 43.19 | 48.7 | 45.88 |
| F7 | 51.5 ± 6.4 | 51.21 | 51.6 | 54.54 |
| F8 | 50.9 ± 4.4 | 49.51 | 48.4 | 44.83 |

**
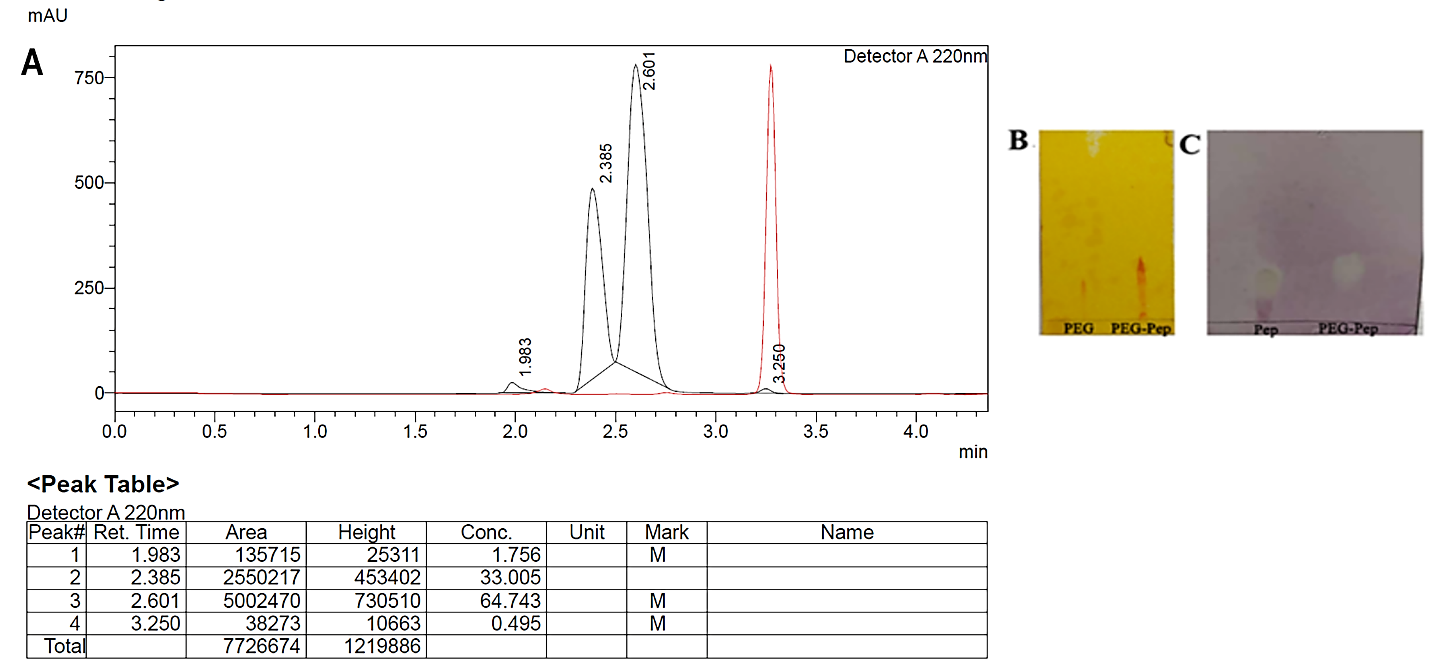
**

Fig. S1 (A) HPLC analysis data for the free MMP-2 cleavable peptide with a retention time of 3.25 min and the mPEG-peptide-DOPE conjugate with a retention time of 2.6 min revealed the consumption of free peptide in the reaction and formation of the conjugate. B) Dragendorff's reagent and C) Ninhydrin reagent staining of the PEG chains and the peptide

**
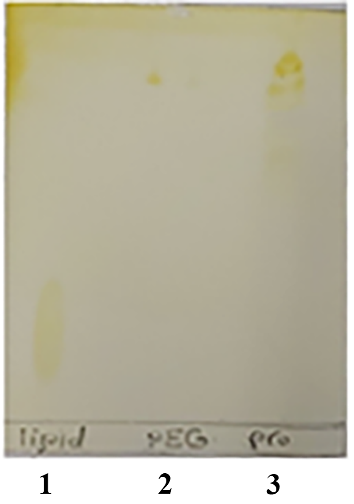
**

Fig. S2 Final product was confirmed by iodine staining of the TLC plate. (1) DOPE, (2) PEG, (3) m-PEG-peptide-DOPE.


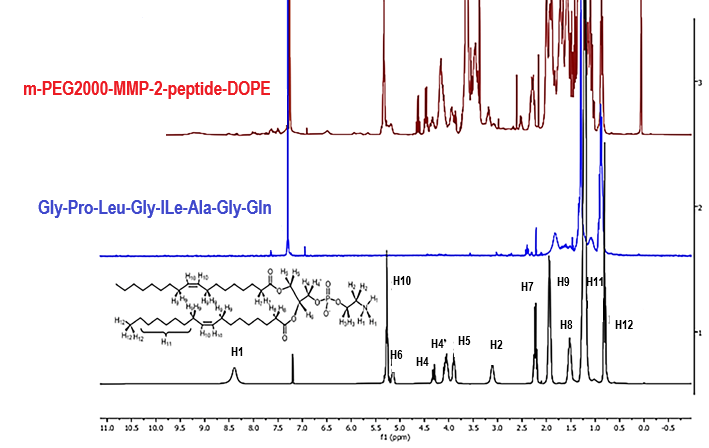


Fig. S3 ^1^HNMR spectra of the DOPE (black), MMP-2 cleavable peptide (blue) or the PEG-peptide-DOPE conjugate (reddish-brown).


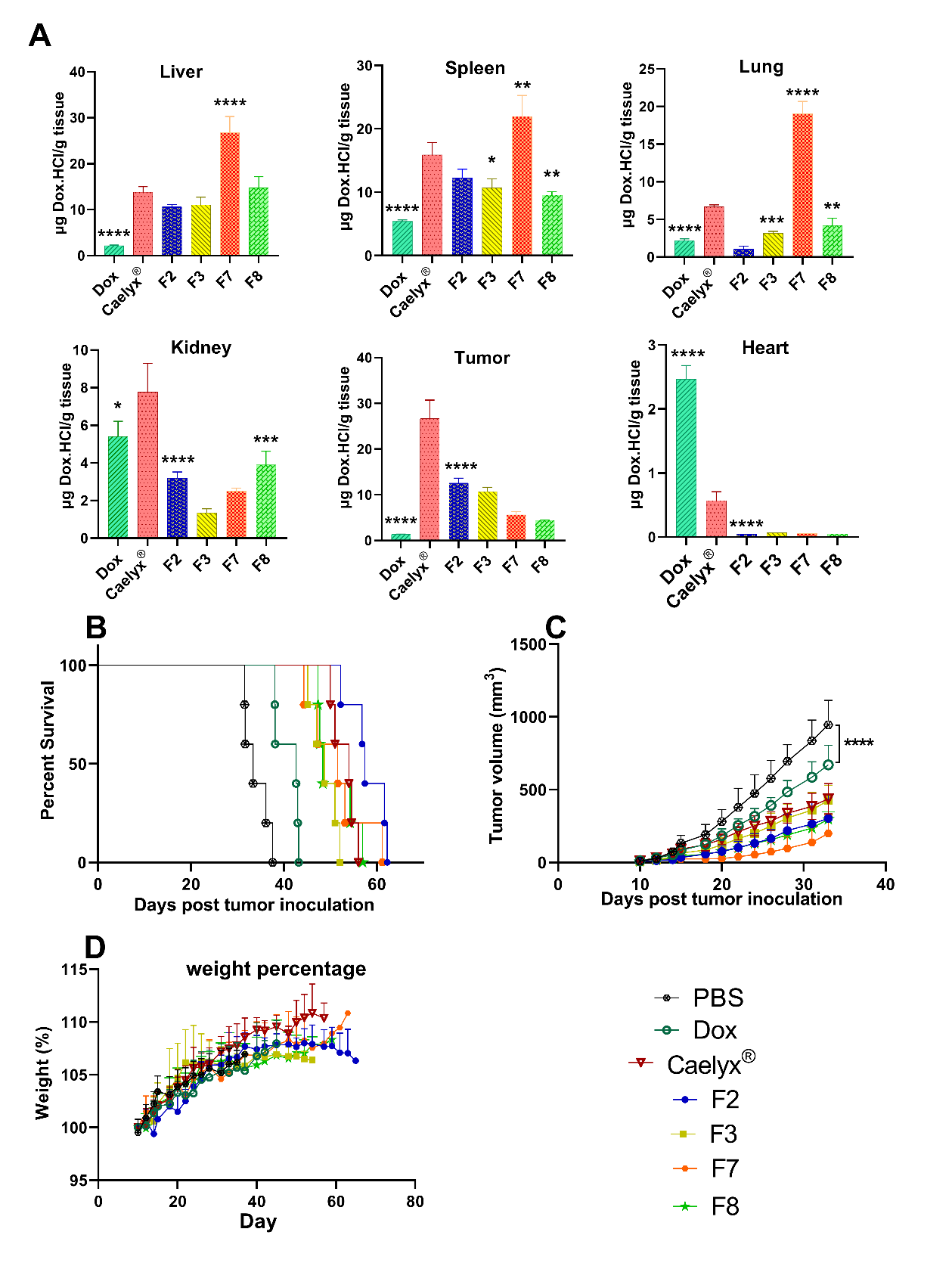


Fig. S4 Biodistribution of the free Dox and Dox-loaded liposomes after 24 h (A) and their therapeutic efficacy in male BALB/c mice bearing C26 tumor [survival curve (B), tumor volume (C) and body weight (D)].

**
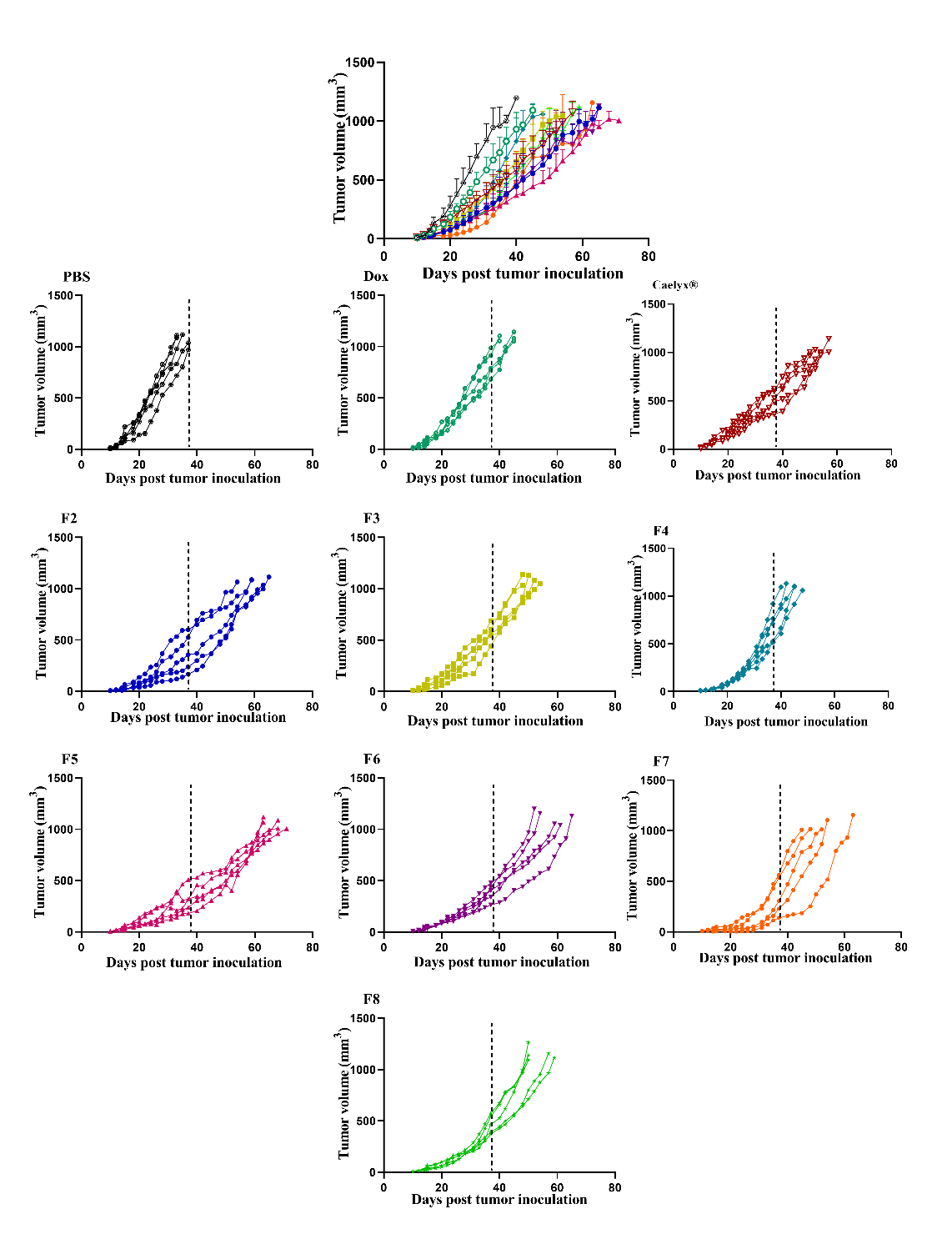
**

Fig. S5 Tumor volume of the C26-bearing mice treated with different liposomal formulations

**References**

Karimi, M., Gheybi, F., Zamani, P., Mashreghi, M., Golmohammadzadeh, S., Darban, S. A., et al. (2020). Preparation and characterization of stable nanoliposomal formulations of curcumin with high loading efficacy: In vitro and in vivo anti-tumor study. *International journal of pharmaceutics*, 580, 119211.

Livak, K. J., & Schmittgen, T. D. (2001). Analysis of relative gene expression data using real-time quantitative PCR and the 2− ΔΔCT method. *methods*, 25(4), 402-408.

Mirhadi, E., Mashreghi, M., Askarizadeh, A., Mehrabian, A., Alavizadeh, S. H., Arabi, L., et al. (2022). Redox-sensitive doxorubicin liposome: a formulation approach for targeted tumor therapy. *Scientific reports*, 12(1), 1-17.

Mirzavi, F., Barati, M., Vakili-Ghartavol, R., Roshan, M. K., Mashreghi, M., Soukhtanloo, M., et al. (2022). Pegylated liposomal encapsulation improves the antitumor efficacy of combretastatin A4 in murine 4T1 triple-negative breast cancer model. *International Journal of Pharmaceutics*, 613, 121396.

Nik, M. E., Malaekeh-Nikouei, B., Amin, M., Hatamipour, M., Teymouri, M., Sadeghnia, H. R., et al. (2019). Liposomal formulation of Galbanic acid improved therapeutic efficacy of pegylated liposomal Doxorubicin in mouse colon carcinoma. *Scientific reports*, 9(1), 1-15.

Riahi, M. M., Sahebkar, A., Sadri, K., Nikoofal-Sahlabadi, S., & Jaafari, M. (2018). Stable and sustained release liposomal formulations of celecoxib: In vitro and in vivo anti-tumor evaluation. *International journal of pharmaceutics*, 540(1-2), 89-97.
